# CO_2_ conversion to CO *via* plasma and electrolysis: a techno-economic and energy cost analysis†

**DOI:** 10.1039/d4ee00164h

**Published:** 2024-05-06

**Authors:** Jose Osorio-Tejada, Marc Escriba-Gelonch, Rani Vertongen, Annemie Bogaerts, Volker Hessel

**Affiliations:** a School of Engineering, University of Warwick UK volker.hessel@adelaide.edu.au; b Department of Environment, Soil Sciences and Chemistry, University of Lleida Spain; c Research Group PLASMANT, Department of Chemistry, University of Antwerp Belgium annemie.bogaerts@uantwerpen.be; d School of Chemical Engineering, University of Adelaide Australia

## Abstract

Electrification and carbon capture technologies are essential for achieving net-zero emissions in the chemical sector. A crucial strategy involves converting captured CO_2_ into CO, a valuable chemical feedstock. This study evaluates the feasibility of two innovative methods: plasma activation and electrolysis, using clean electricity and captured CO_2_. Specifically, it compares a gliding arc plasma reactor with an embedded novel carbon bed system to a modern zero-gap type low-temperature electrolyser. The plasma method stood out with an energy cost of 19.5 GJ per tonne CO, marking a 43% reduction compared to electrolysis and conventional methods. CO production costs for plasma- and electrolysis-based plants were $671 and $962 per tonne, respectively. However, due to high uncertainty regarding electrolyser costs, the CO production costs in electrolysis-based plants may actually range from $570 to $1392 per tonne. The carbon bed system in the plasma method was a key factor in facilitating additional CO generation from O_2_ and enhancing CO_2_ conversion, contributing to its cost-effectiveness. Challenges for electrolysis included high costs of equipment and low current densities. Addressing these limitations could significantly decrease production costs, but challenges arise from the mutual relationship between intrinsic parameters, such as CO_2_ conversion, CO_2_ input flow, or energy cost. In a future scenario with affordable feedstocks and equipment, costs could drop below $500 per tonne for both methods. While this may be more challenging for electrolysis due to complexity and expensive catalysts, plasma-based CO production appears more viable and competitive.

## Abstract 2

Analysis of the CO production cost from CO_2_*via* gliding arc plasma reactors with embedded carbon beds versus low-temperature electrolysers.

## Graphical abstract

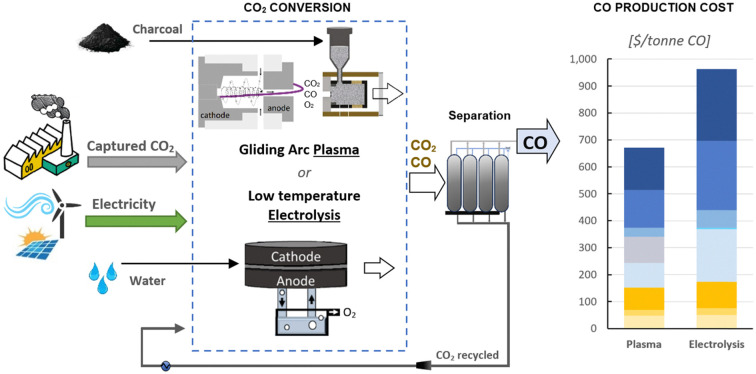

**Broader context**

Carbon monoxide (CO), a crucial feedstock for various chemical products, is primarily derived from the carbon content of fossil fuels using thermochemical methods. Nevertheless, with advancements in and the competitiveness of clean electricity and carbon capture technologies, methods such as plasma activation and electrolysis have emerged as vital tools for decarbonizing the chemical industry. While advances in fundamental research have demonstrated potential for converting CO_2_ to CO *via* plasma and electrolysis, comprehensive techno-economic evaluations for commercial viability remain limited. Existing studies on the economics of electrolysis-based methods often rely on assumptions and parameter values combined from studies conducted under different experimental conditions, thereby affecting the reliability of the results. In the realm of plasma-based CO_2_ conversion, economic studies are even more limited, CO production cost in a gliding arc plasmatron and the impact of embedding a carbon bed within a plasma reactor have not been analysed. Recognising these gaps, our study contrasts the economic viability of CO production *via* plasma and electrolysis, utilizing our own actual data and individual studies conducted under specific experimental conditions. Through sensitivity analyses, considering the mutual relationship between the various operating parameters, we identify the critical aspects for deploying these alternative technologies.

## 1 Introduction

Decarbonising the chemical industry through electrification and implementing carbon capture and utilisation technologies, are two main strategies toward the net-zero emissions scenario by 2050.^1,2^ One of the pathways to this aim is the use of technologies for carbon dioxide (CO_2_) conversion to carbon monoxide (CO), a valuable feedstock for different products. The major uses of CO include the preparation of syngas (a mixture of CO and H_2_) to produce commodity chemicals at a very large scale, such as methanol. Other uses of CO include the preparation of acetic acid, formic acid, acrylic acid, propanoic acid, dimethylformamide, and acetic anhydride.^3^ CO is also used on smaller scales in metallurgy, in plastics industries by reacting CO with chlorine to produce phosgene, and in medical, pharmaceutical, electronics, and other specialty chemicals industries.^4^

For large-scale applications, CO is usually produced on-site *via* thermochemical methods, such as reforming, partial combustion, or gasification plants, using fossil resources, and utilized locally within the plant.^4^ However, for smaller-scale applications, on-site CO production becomes costly, making it necessary to purchase CO stored in cylinders or tube trailers. This process is hazardous, and expensive when transported over long distances due to its toxicity and flammability concerns.^3,4^ CO is traditionally produced on a very large plant at a cost of approximately $300 per tonne.^5,6^ In contrast, high-purity CO cylinders can cost up to $3000 per tonne.^6^

Two alternative technologies for CO production are CO_2_ electrolysis and CO_2_ activation by plasma, which operate with electricity and captured CO_2_. These electrochemical methods show potential for commercial-scale implementation in the next years. However, since CO derivatives such as methanol can be produced for less than $1 per kg *via* current industrial processes, the implementation of emerging CO_2_ conversion technologies in these large industries would be hardly profitable, regardless of their performance.^7^ Hence, the penetration of plasma- and electrolysis-based CO production in the market would be more feasible for small-scale applications that require flexible, modular, and on-demand CO supply.^8^ This decentralised CO production reduces the safety risks and costs associated with on-site CO storage and transport, when cheaper and safer transported CO_2_ is used as the feed gas. Besides these advantages, these technologies offer an easy start/stop operation, multiple sizes, high purity options, and lower costs related to storage, rentals, and connections, enabling the decoupling of the production of chemical products from fossil fuels.

### 1.1 CO_2_ conversion by plasma-based technologies

Plasma is an ionised gas, containing various radicals, excited species, ions, and electrons. When a plasma is generated by applying an electric field, the electrons are more efficiently heated by the applied electric power than the other species, thereby activating stable gas molecules such as CO_2_ through electron impact reactions. This technology has been demonstrated in various industrial applications, such as the removal of volatile organic compounds,^9^ ozone production,^10^ and arc plasma furnaces for steelmaking.^11^ More recently, this technology has been increasingly investigated for sustainable applications, such as N_2_ fixation for fertiliser production^12–14^ and CO_2_ conversion into value-added chemicals.^15–17^

The performance of plasma-based CO_2_ conversion to CO is characterized by several common intrinsic parameters.^18,19^ Among these parameters (and assumed ranges) are the operating pressure (from a few Pa to atmospheric pressure); the input flow rate (1000–100 000 cm^3^ min^−1^), which is determined by mass flow controllers; the plasma power (1–10 kW), which is the true power that is dissipated in the plasma; the specific energy input (2–1000 MJ m^−3^), which reflects the amount of energy deposited by the plasma per volume or molecule of gas; the absolute conversion (ranging from 0.5–80%, for different types of plasma reactors), which represents the percentage of reactant converted per total reactant input; and the energy efficiency (1–80%, again depending on the plasma reactor) which is a measure of how efficiently the plasma conversion process performs compared to the standard reaction enthalpy.^16,18^

Another parameter to influence the CO_2_ conversion in plasma is the addition of a catalyst as studied in the field of plasma catalysis.^20^ More research is needed to understand the complex interactions between the plasma and the catalyst; therefore, these parameters will not be considered for the analysis in this work.

In the context of large-scale implementation, other intrinsic parameters play a role that are not widely reported yet. For example, the equilibration time is usually not reported but is typically assumed to be in the order of (milli)seconds.^6^ Some plasma reactors, however, require more extensive ignition procedures, such as the transition from low to atmospheric pressure (as in some microwave (MW) plasmas), which is important in the context of the start/stop operation. The plug-to-power efficiency – or the accounting of energy lost in the power supply unit due to resistive losses in transformers, rectifiers, and conversion stages – is another relevant intrinsic parameter for efficiency.^21^ This parameter largely depends on the exact coupling between the plasma and the power supply, although this might vary with the scale since large-scale power supplies are optimized for one type of plasma reactor. Still, the experimental plug-to-power efficiencies can be a better indication of the real efficiency compared to the plasma power, certainly for industrial applications. Specifically, the efficiencies of our AC–DC power units range from around 80% to as low as 60%, as measured in multipurpose power units in our lab. The lifetime and stability of the electrodes are also important for commercialization. While this is not an issue in MW plasmas and dielectric-barrier discharge (DBD) reactors, due to the electrodeless configuration and dielectric layer, respectively, some gliding arc (GA) plasma reactors show slight damage at the electrodes.^22^ This damage is still significantly smaller than in thermal plasma applications, and therefore, the effect on the performance is generally assumed to be very small.^23^ The advantage is that a plasma reactor does not require rare metal catalysts for good performance. The reactions take place in the entire plasma phase and are not limited by mass transfer to a surface, meaning that electrode damage is less important compared to electrolysis. This mass transfer limitation might play a role in the field of plasma catalysis where the catalyst is placed inside the discharge, but the latter is out of scope for this work, since it is more applicable for complex reaction mixtures such as dry reforming of methane, to tune the selectivity.^20,24^

The possible range of parameters and performance largely depends on the type of plasma reactor. However, it remains challenging to compare these reactors, since they have distinct advantages depending on the research focus. DBD reactors are common due to their simple design, relatively low gas temperature, and atmospheric pressure operation, which makes them interesting to study plasma catalysis.^24^ High conversions have been reached (up to 75%), but the energy efficiency remains limited (typically <10%, exceptionally up to 20%).^25,26^ MW reactors have an electrodeless configuration, which increases the lifetime of these reactors, and their quartz reactor tubes facilitate *in situ* diagnostics. They reach higher efficiencies (up to 50%)^27^ thanks to the high temperature and reduced pressure, but the vacuum pump is usually not included in the energy efficiency. Another plasma type that operates at atmospheric pressure with good energy efficiency (about 40%), is the GA reactor, although its conversion remains limited (up to 15%). They are easy to operate and allow for various reactor designs, such as 2D gliding arc, rotating gliding arc, gliding arc plasmatron (GAP), magnetic gliding arc, *etc.*^28–31^

In general, a trade-off exists between conversion and efficiency for plasma-based CO_2_ splitting, *i.e.*, a higher specific energy input enhances the CO_2_ conversion but often reduces the energy efficiency. Moreover, the overall performance has not improved drastically when comparing the review of Snoeckx and Bogaerts (2017)^16^ to the recent work of Vertongen and Bogaerts (2023).^18^ They suggest other routes for improvement beyond the plasma reactor, such as a carbon bed^32–37^ and quenching in the post-plasma zone.^38^ However, the typical lab-scale experiments remain insufficient to draw conclusions on the economic feasibility of plasma technology at an industrial scale. Therefore, a techno-economic analysis (TEA) is the crucial next step to investigate the real-world application.

A few studies have explored the economic aspects of plasma-based CO production. For instance, van Rooij *et al.* (2018) proposed a plant with linear upscaling of MW plasmas,^6^ while Detz and van der Zwaan (2022) examined the influence of unit size and learning rate on the uptake of this technology.^39^ However, these examples focused on the process design and only analyse the minimum selling price of CO. Lamberts-Van Assche *et al.* (2022) define a more general process where only the DBD plasma reactor is modelled in the foreground system, highlighting the electricity price and power supply investment as significant cost factors.^5^ All these examples conclude that plasma-based CO production is not yet competitive, emphasizing the need for substantial improvements in conversion and reductions in electricity costs. While other plasma applications, such as nitrogen fixation for various chemicals,^40–43^ air pollutants removal^44^ and landfill waste treatment,^45^ initially demonstrated limited competitiveness in early economic studies, subsequent advancements have aligned with predictions, nearing industrial use.^46–49^ These diverse applications underscore the critical role of early economic analysis in advancing and deploying novel plasma technologies.

### 1.2 CO_2_ conversion by electrolysis-based technologies

The study of electrolysis-based CO_2_ conversion, also known as CO_2_ reduction (CO2R), has exponentially increased over the past decade, with the number of publications rising from less than 200 in 2010 to over 2000 in 2020.^50^ Much of this inquiry has been conducted at a lab scale, focusing on reactor and catalyst development, atomic and molecular modelling, and reaction kinetics, primarily centred on low-temperature electrolysers.^51^

Other technologies for CO2R include molten carbonate electrolysis cells (MCEC) and solid oxide electrolysis cells (SOEC), which operate at temperatures between 500 and 900 °C,^3,52^ resulting in favourable thermodynamics and reaction kinetics.^53^ Among these technologies, SOEC stands out for its higher maturity stage. SOEC systems for CO2R have been demonstrated at stack and system levels,^3^ reaching commercial availability, such as the eCOs™ product by Haldor Topsoe, offered as a reliable on-demand device for CO production.^54^ However, the distinct operational characteristics of SOEC and MCEC render direct comparisons with low-temperature electrolysis- and plasma-based technologies challenging, due to their differing product functionalities. High-temperature operation results in longer startup and load response times, requiring time to reach the appropriate temperature for a steady-state process.^55^ In contrast, low-temperature processes can be shut down and started up in milliseconds due to low thermal inertia,^16^ making them better suited for intermittent electricity supply or irregular production needs. Consequently, only low-temperature electrolysis technologies would offer comparable functionalities to plasma-based units.

Research on TEA of CO2R has been limited, although it has gradually gained attention in the last few years.^7,8,56–65^ The majority of these TEA have focused on the CO2R to C_1_–C_2_ chemicals. Collectively, it is acknowledged that CO production might be competitive in scenarios with specific extrinsic and intrinsic parameters. For extrinsic parameters, we have observed that most authors have assumed low electricity prices, typically around 0.025 $ per kW h. There is also a high dispersion in the assumed costs of CO_2_ feedstock, ranging from 17 to 70 $ per tonne, and electrolyser cells, with costs between 920 and 20 000 $ per m^2^.

When considering the data used for intrinsic parameters (and their assumed ranges), the following figures are of importance: operating cell voltage (2.0–2.5 V), which reflects the amount of energy required for the conversion; current density (110–500 mA cm^−2^), defined as the current flow rate divided by the area of the electrode, which denotes the overall productivity or the catalyst activity; faradaic efficiency (FE) (70–100%), used to express the selectivity of an electrocatalytic reaction as the fraction of the total charge used for the product formation; single-pass conversion (SPC) (10–50%), which represents the percentage of reactant converted per total reactant input, meaning that a process with low SPC delivers a gas mixture with a high amount of unreacted CO_2_.

Other relevant intrinsic parameters for CO2R are not yet reported at the scale needed for commercial implementation, such as the process stability and the electrolyser size. Stability refers to the ability to maintain constant selectivity and activity. This is related to the durability of membranes, electrodes, and catalysts,^51^ which results in an increase in cell voltage and a decrease in the FE in the long run, negatively affecting energy costs. It has also been observed that SPC values can decrease after a few hours, depending on the catalyst type and loading.^66^ In this sense, it is believed that maintaining a stable operation for at least 5000 hours is necessary for commercial implementation.^67^ However, most reports indicate brief testing periods, typically less than 100 hours.^60^ Further long-term experimental research is required to improve stability, leading to reduced costs for maintenance, component replacement, and associated downtime.^68^ Another underexplored aspect is the optimal size of the electrolyser. Although water electrolysers are more commercially developed, it is uncertain if the successful designs in those systems will also be the most effective for CO2R due to substantial differences in their chemistries.^50^

Furthermore, as seen in plasma research, plug-to-power efficiency is not frequently reported in CO2R-related publications. Previous TEA studies have not mentioned the specific plug-to-power efficiencies,^7,8,56–65^ suggesting that they may have overlooked these losses by estimating the power needed solely based on the DC voltage and current required in the electrolysis cell. This could lead to an underestimation of the actual electricity costs, considering that commercial water electrolyser systems can experience AC–DC conversion energy losses of about 10%, along with an additional 8% or more in energy losses due to consumption in auxiliary instruments and non-usable energy associated with power levels above or below the electrolyser operating limit.^69,70^

The inconsistent reporting of key parameters is primarily due to the research focus on material design and limited attention to reactor engineering and scale-up efforts,^71^ which are essential for addressing stability, size, and SPC challenges. Consequently, assumptions regarding these parameters must be made for economic analyses. No constraints on process stability and similar sizes to commercial water electrolysis systems are typically assumed. However, for SPC, the assumed value is either not mentioned, set without a clear reference, or based on the few reported values in the literature. Jouny *et al.* (2018) chose an SPC of 50% and assumed a high FE of 90% with a current density of 200 mA cm^−2^ and a cell voltage of 2.3 V.^7^ The authors also mentioned that SPC values are infrequently reported in the literature, which are typically below 10%, but one study reported values near 35%.^72^ Yet, in this study, an input gas mix with 90% N_2_ was used. This mixture enhanced the CO_2_ conversion but resulted in a lower current density of 29 mA cm^−2^, an FE of 85%, a higher cell voltage of 3.0 V, and an output gas containing a large amount of N_2_ that requires separation. When pure CO_2_ input was considered, the current density increased to 51 mA cm^−2^, but the SPC decreased to 5%.

Similarly, Ramdin *et al.* (2021) assumed a 50% SPC for the base case with a high FE of 95% at a current density of 300 mA cm^−2^ and a cell voltage of 2.5 V.^64^ The assumed SPC was based on O’Brien *et al.* (2021) who stated that the fundamental conversion limit in anion exchange membrane electrolysers is 50%.^73^ However, in practice, high conversions are obtained at very low flow rates. It has been demonstrated that the SPC is reduced as the flow rate is increased.^66,73–75^ Moreover, Ren *et al.* (2019) also found that when flow rates are increased, the FE increased up to 99%, but the SPC decreased to almost zero.^75^ Additionally, the fundamental conversion limit is affected by the CO_2_ loss due to (bi)carbonate formation and CO_2_ crossover (*i.e.*, unreacted CO_2_ molecules escape from the cathode by crossing the membrane or electrolyte and reaching the anode), which generally results in an SPC lower than 30%.^56,73^ Yue *et al.* (2022) also noticed that SPC has received little attention so far and that a few studies have obtained values usually lower than 10%, especially at high current densities.^56^

For these reasons, it would be inappropriate to adopt SPC values based on fundamental limits or by combining various studies different than the ones where the other optimal parameters were obtained. This is due to the diverse experimental conditions that can lead to optimal FE, cell voltage, current densities, and flow rates for industrial applications, while delivering very low SPC values. Therefore, combining process parameters for a TEA from different reports would affect the reliability of the results. This is particularly important because the downstream separation costs usually represent a large portion of the total production costs of chemicals.^68^ Therefore, assuming high SPC values would lead to an underestimation of the total CO production cost. For this reason, further research and reporting on SPC values are required to evaluate and commercialize CO2R.^8,68^

In various TEA studies, researchers have set intrinsic parameters close to the optimal values for cost-effective implementation, and some of them have also conducted sensitivity analyses by varying specific parameters. Kibria *et al.* (2019) concluded that feasible commercial systems would need current densities above 300 mA cm^−2^, FE over 80%, and cell voltages below 1.8 V.^60^ Masel *et al.* (2021) recommended current densities up to 500 mA cm^−2^ because operation above this limit leads to overheating and problems with water management from the high required voltages.^51^ Therefore, as the electrolyser costs continue to decrease, lowering cell voltage for reduced energy costs takes precedence rather than achieving high current densities for capital cost reduction. This approach also helps to maintain acceptable process selectivity, as numerous studies indicate that FE is frequently low at current densities over 200 mA cm^−2^.^3,76,77^ Yue *et al.* (2022) also concluded that the best strategy is decreasing the cell voltage.^56^ This was because the study assumed a high electricity price of 0.04 $ per kW h and a very low electrolyser price of 920 $ per m^2^. If an electrolyser price of 10 000 $ per m^2 ^^64^ and a lower electricity price had been assumed, the best strategy would be to increase the current density. This parameter has been the most important in other studies due to the high capital costs of operating at low current densities, compared to commercial water electrolysers that achieve current densities over 1000 mA cm^−2^ at moderate overvoltages with FE over 90%.^67,78^ However, achieving high current densities is challenging for CO2R due to significant overpotentials and low FE.

For these reasons, it becomes essential to comprehensively address the complexity of varying parameters in CO_2_ conversion. This requires an understanding of how changes in one parameter can affect the other parameters. Moreover, the values defined for the base cases should ideally be derived from experimental studies that report metrics for all required parameters. Relying on such studies helps to avoid assumptions that could compromise the reliability of the results.

In essence, our study aims to perform an exhaustive TEA for plasma- and electrolysis-based CO production, utilising our own data and individual studies conducted under given experimental conditions. This approach sets itself apart from existing literature, which often relies on mixed data sets and overlooks the mutual relationship between the various operating parameters of the evaluated technologies. Furthermore, this work represents the first endeavour to analyse CO production costs in a GAP and to assess the economic impact of incorporating a carbon source on enhancing CO yield, a factor that bears significant implications.

## 2 Methods

### 2.1 Process design

#### 2.1.1 Plasma-based CO_2_ conversion

**Fig. 1:**
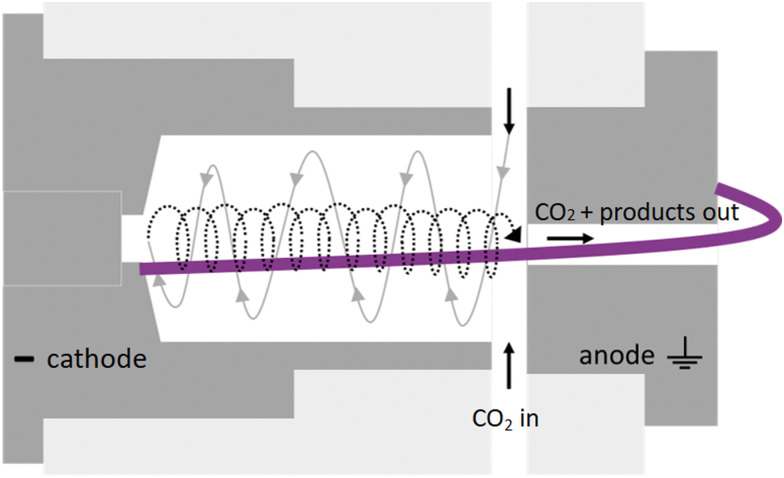
Schematic 2D representation of the basic GAP reactor. Dark grey indicates the cathode and anode electrodes, and light grey represents how they fit into the reactor body. The white space represents the gas volume. The left- and rightward vortex of the gas are schematically represented in grey and black, respectively, and an artistic presentation of the arc is given in purple. The gas flows in through six tangential inlets from the sides, goes inside the reactor volume, and then flows out through the anode.

We selected the operating parameters for plasma-based CO_2_ conversion based on experimental results in our previous work.^18^ The experiment applies an optimized GAP reactor, as depicted in Fig. 1.

Its characteristic reverse vortex flow limits the heat losses of the plasma to the walls and can enhance CO_2_ conversion by mixing the hot plasma core and surrounding cooler gas. The intrinsic parameters are summarized in Table 1 and compared to typical values of other reactor types for CO_2_ conversion.

**Table 1:** Intrinsic parameters of the GAP, considered in this TEA, and comparison with various other plasma types for CO_2_ conversion at atmospheric pressure

Authors	Type of plasma reactor	CO_2_ feed rate (cm^3^ min^−1^)	Plasma power (W)	Specific energy input (MJ m^−3^)	Energy efficiency (%)	Single pass conversion (%CO_2_)
Vertongen and Bogaerts (2023)^18^	GAP	10 000	835	3.85	29	9.65
Girard-Sahun *et al.* (2022)^32^	GAP	10 000	530	3.2	27.9	7.6
Girard-Sahun *et al.* (2022)^32^	GAP^a^	10 000	530	3.2	45.4	12.6
Uytdenhouwen *et al.* (2019)^26^	μ-DBD	1.5	30	1200	0.74	70
Ozkan *et al.* (2017)^25^	DBD	200	50	15	20	26
Bongers *et al.* (2017)^80^	MW^b^	11 000	8000	44	22	83
Mitsingas *et al.* (2016)^27^	MW	4000	150	2.25	50	6

a Results after embedding a carbon bed.

b Reaction performed at 20 kPa.

The plasma reactor outlet stream is a mixture of unconverted CO_2_ (90.35 wt%), CO (6.14 wt%), and O_2_ (3.51 wt%), based on the single-pass conversion presented in the first row of Table 1. The separation of the CO product from this mixture is not a straightforward or low-cost process. At present, no mature processes exist for the separation of CO from this kind of mixtures, due to the presence of O_2_.^6^ A two-step separation process was proposed by van Rooij *et al.* (2018), in which the CO_2_ content is first removed, and then CO is separated from O_2_ using a pressure swing adsorption (PSA) unit.^6^ However, the authors concluded that the proposed separation process became a dominant factor in the total CO manufacturing costs and suggested focusing efforts on preventing the O_2_ generation during the CO_2_ conversion stage. Similarly, Kaufmann *et al.* (2023) proposed a system for O_2_ removal based on gas diffusion electrodes (GDE) to be implemented after a plasma reactor.^79^ This GDE-based separation process, besides its high energy requirements, also accounted for 70% of the total capital costs of the plant.

For these reasons, recent investigations have proposed a novel approach for removing O_2_ from the outlet streams of plasma reactors by utilising embedded carbon beds.^32,33,36,37^ The mechanism incorporates a solid carbon source, such as activated charcoal or biochar, into a basket inside the reactor and next to the stream outlet. The O atoms and O_2_ molecules generated after initial CO_2_ dissociation reaction (CO_2_ → CO + O, or CO_2_ → CO + ½O_2_) react with the incorporated carbon to form additional CO product (C + O → CO, or 2C + O_2_ → 2CO). Experiments have demonstrated that the carbon bed also enhances the SPC through two mechanisms. Firstly, the rapid consumption of the generated O/O_2_ could prevent the recombination of O/O_2_ with CO. This recombination leads to the formation of CO_2_, resulting in a decline in the high conversion achieved during the early stages of the plasma reaction. Secondly, a portion of the CO_2_ feed could react with C to produce more CO *via* the reverse Boudouard reaction (CO_2_ + C → 2CO). Girard-Sahun *et al.* (2022) investigated the underlying mechanisms of both reactions using a kinetic model.^32^ However, as noted by Huang *et al.* (2021), the precise contributions of these mechanisms to the final SPC and CO concentration remain unclear.^33^ Nonetheless, promising results obtained from experiments and modelling conducted by the PLASMANT research group have shown that embedding a carbon bed can increase the SPC by 66% and triple the CO concentration, although these improvements were only obtained for measuring times of a few minutes.^32^ Current efforts within the PLASMANT group, however, indicate that this enhancement can be maintained over longer time-scales by optimizing the carbon bed and carbon supply system.

**Fig. 2:**
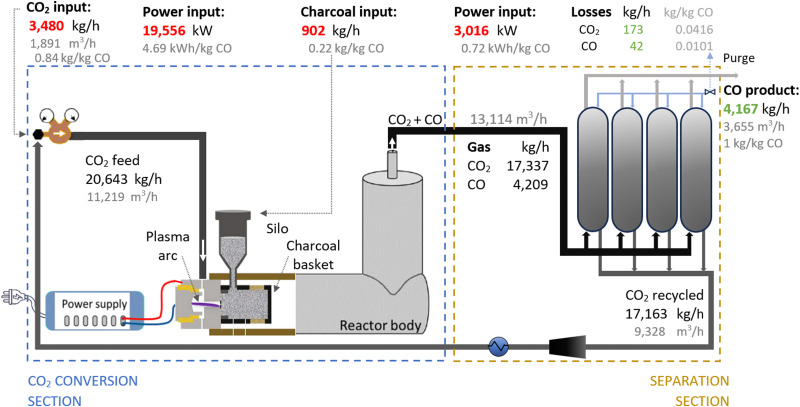
Schematic flowchart for the plasma-based plant, producing 100 tonnes CO per day. Carbon bed design based on Girard-Sahun *et al.* (2022).

Therefore, we proposed the plasma-based CO production plant with embedded carbon bed, delivering a 16.02% SPC (*i.e.*, 9.65% (*cf.*Table 1) + 66% increase due to a carbon bed), detailed in Fig. 2. The carbon bed is provided with an innovative silo system, which constantly supplies the material to avoid carbon depletion upon reaction with O/O_2_. For this process design, we assumed that the SPC increase—specifically, the additional 6.37% of CO_2_ converted—was entirely due to the reverse Boudouard reaction. This is because it is not possible to estimate the extent to which the increase was due to the prevented recombination of O/O_2_ with CO. Furthermore, we assumed that the initially generated O_2_ from the plasma-based CO_2_ dissociation (9.65% SPC) was converted to CO. Based on these two reactions, we calculated the required solid carbon input, assuming complete material consumption.^32,33,37^

A plant with a production capacity of 4167 kg CO per h (*i.e.*, 100 tonne CO per day) was selected, as this is the typical demand evaluated in previous analysis for alternative CO production.^8,59^ We assumed that the performance of the experimental setup is maintained in the upscaled system by setting up identical reactors in parallel (upscaling challenges are addressed in Section 2.1.3). Based on the defined parameters, the power measured to run the plasma arc was 15 645 kW, equivalent to 3.75 kW h per kg CO. However, assuming an 80% plug-to-power efficiency due to energy losses in the AC–DC power supply unit required to convert the AC energy from the grid or wind power farms, and considering no extra energy was required to run the carbon bed, the applied power was about 19 556 kW. This translates to an energy cost for the CO_2_ conversion section of 4.69 kW h per kg CO. From this section, the reactor output stream consisted of unconverted CO_2_ (80.47 wt%) and CO (19.53 wt%), with all O_2_ removed, which requires simpler separation systems.

To separate the CO product from the unconverted CO_2_, PSA units are commonly used in industrial processes with similar gas compositions. They are also preferred for treating the gas output streams in electrolysis-based CO_2_ conversion systems due to their high efficiency and cost-effectiveness.^7,56,60,81–84^ In this sense, we assumed the same separation system for both compared plants, which allowed us to focus the analysis on the core sections of CO_2_ conversion. For this separation section, a power of 3016 kW is required to treat 13 114 m^3^ h^−1^ of gas. This is considering an energy consumption of 0.23 kW h m^−3^ input gas, based on Paturska *et al.* (2015),^85^ a value also assumed in previous studies for CO2R using PSA systems.^7,56,59,60,84^ The separated CO_2_ is recycled back into the reactor feed. During the product separation process, 1% of all products are lost.^64^

#### 2.1.2 Electrolysis-based CO_2_ conversion

**Fig. 3:**
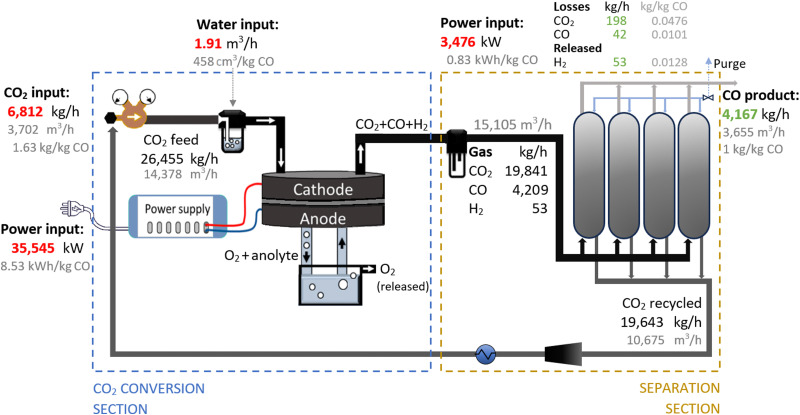
Schematic flowchart for the electrolyser-based plant, producing 100 tonnes CO per day.

In selecting the operating parameters for the analysis, we focused on studies reporting experimentally obtained results that utilized a pure CO_2_ feed, *i.e.*, not diluted with N_2_ mixtures, and conducted at atmospheric conditions. Moreover, we only considered those studies reporting all the necessary parameters to perform the TEA, including feed rate, cell voltage, current density, FE, and SPC. Almost all publications on CO2R reported values for the first four parameters, but just a few reported the SPC value, which are those included in Table 2. For each study, we presented the results for the maximum CO_2_ feed rate tested with sustained operation. Given the importance of stability and scalability of the system (further discussed in Section 2.1.3), we selected the intrinsic parameters obtained in the study performed at 1000 cm^3^ min^−1^ for long runs by Endrődi *et al.* (2019).^74^ We chose these values over other more favourable options, such as the high current density achieved by Bhargava *et al.* (2020),^89^ as their results corresponded to short runs and used a very small feed rate of 17 cm^3^ min^−1^, requiring a significantly elevated quantity of devices in parallel to reach the required production volume. For the defined system, the energy required by the electrolysis cells is 6.82 kW h per kg of CO, which translates to a total requirement of 8.53 kW h per kg of CO, assuming an 80% plug-to-power efficiency. The schematic description of the process is presented in Fig. 3.

**Table 2:** Intrinsic parameters of various CO2R technologies at atmospheric pressure

Authors	Type of electrolyser	CO^2^ feed rate (cm^3^ min^−1^)	Cell voltage (V)	Current density (mA cm^−2^)	Faradaic efficiency (%)	Single pass conversion (%CO_2_)	Electrolyte	Cathode catalyst		Anode catalyst	
								Metal	Loading (mg cm^−2^)	Metal	Loading (mg cm^−2^)
Endrődi *et al.* (2019)^74^	Zero gap^a^	1000	3.0	250	85	25	1.0 M KOH	Silver	3.0	Ir on a Ti frit	1.0
Jeanty *et al.* (2018)^86^	Three compartment^d^	200	6.0	150	53	23	0.4 M K_2_SO_4_	Silver	NS	NS	NS
Endrődi *et al.* (2021)^87^	Zero gap^b^	100	3.2	420	90	23	H_2_O vapor	Silver	1.0	Ir on a Ti frit	1.0
J. Lee *et al.* (2021)^61^	MEA	75	2.2	223	93	64	1.0 M KOH	Silver	2.0	Nickel	NS
Endrődi *et al.* (2020)^88^	Zero gap^c^	25	3.4	700	90	29	0.1 M CsOH	Silver	1.0	Ir on a Ti frit	1.0
Bhargava *et al.* (2020)^89^	Flow cell	17	3.0	866	98	36	3 M CsOH	Silver	2.0	Iridium oxide	3.0
Kim *et al.* (2015)^72^	Flow cell	7	3.0	51	83	5	1 M KCl	Silver	0.9	Platinum	1.0

a Zero-gap type electrolyser with a multi-stack configuration with 3 cells in series.

b To obtain these results using pure water as anolyte, the cathode needs to be activated by periodically infusing the cathode with different alkali cation-containing solutions.

c The current density starts near 1000 mA cm^−2^, but it stabilises around 700 mA cm^−2^ after 2 hours of operation.

d Three compartment gas diffusion electrode (GDE); NS: not specified; MEA: membrane electrode assembly.

The considered experimental data did not report on CO_2_ losses due to carbonate formation and CO_2_ crossover to the anode channel. Therefore, as the defined SPC of CO_2_ was 25%, we assumed that the remaining 75% of CO_2_ that is unutilized flowed through the cathode gas output and was recycled back after being separated from the products. Yet, we have considered that 1% of all products are lost in this process.^64^

Given that in reactions with FE and SPC below 100%, a mixture comprising CO_2_, CO, and a small amount of hydrogen is obtained from the cathode, no downstream processing is considered for the generated oxygen (from the anode) and hydrogen, because investing in equipment and energy for purification and storage is not profitable due to the low amount of these by-products.

The potassium hydroxide (KOH) electrolyte helps in the transfer of ions and maintains charge neutrality within the cell. This allows for continuous CO_2_ conversion without significant consumption or degradation of the electrolyte while it is recirculated within the system. Therefore, the impacts of this feedstock are neglected since no consumption is assumed in the reaction. In contrast, the deionised water consumed in the side reactions needs to be fed at a rate of 458 cm^3^ per kg CO.

#### 2.1.3 Challenges in upscaling

The upscaling of plasma reactors and electrolysers must meet industrial production requirements efficiently and economically, but it also faces technical challenges, such as the mixing (mass transfer) and heat transfer performance.^90^ One strategy for upscaling is to increase the volume (scale up) of the reactor used in the lab-scale by (i) sizing up the tubing diameter, or (ii) sizing up the reactor length by adding multiple reactors in series. This approach is not suitable for scaling up plasma-based CO production because, while it increases process efficiency, the process performance would be highly influenced by the geometry of a single reactor. Indeed, the plasma volume does not rise to the same extent as the reactor volume, as the plasma arc is typically concentrated in the centre. Hence, a lot of gas can pass through the reactor without being converted. The most prominent upscaling strategy includes (iii) the numbering up, where the number of linked reactors in parallel is increased while keeping the individual geometry of each;^91^ a concept that has pioneered industrial use of flow chemistry and microreactors. ‘Equalling-up’ of the same reaction zones within one joint reactor shell has been coined ‘internal’ numbering-up, while multiplying the same reaction zones each with its own reactor shell is ‘external’ numbering-up. The latter has more commonly taken in plasma reactors because of the larger reactor volumes (as opposed to micro reaction zones favouring internal assembly). Flow chemistry is also ‘scaled-up’ by accessing unusual, highly productive process regions, coined ‘novel process windows (NPW)’;^92,93^ yet plasma processing itself is already an NPW.

Successful plasma applications in microelectronics, ozone generation, polymer treatment, plasma-assisted ignition and combustion, *etc.* have been described.^94,95^ Key factors for upscaling plasma technology also include power supply, gas handling, safety protocols, and environmental considerations. There are limitations regarding plasma instabilities due to perturbations of temperature, operational pressure, average power density, and reduced electric field. Another constraint when scaling continuous plasma systems, such as industrial ozone generators with DBD plasma, is that the reactors reach a lower power (3 orders of magnitude below the most powerful arcs), for a much larger size of the reactor (at least 3–4 orders of magnitude as compared with thermal plasma).^23^

Yamamoto *et al.* (2007) simulated plasma-based CO production using the numbering-up approach, not meaning necessarily increasing the number of whole reactor components, but one power supply, one anode, one cathode, and *n* sets of discharge tubes, where only the discharge tubes are multiplied.^96^ Different from a conventional external numbering-up, where complete reactors are parallelized, such approach is actually an internal numbering-up strategy, as mentioned above, where multiple microchannels are placed in one reactor.^91^ The latter decreases the total inventory of equipment and consequently the setup footprint and boosting economic feasibility, enabling studies, such as hydrogenations,^97^ fluorinations,^98^ or radical polymerizations.^99^ The payback is the distribution of the reaction stream.

Following this approach and according to the simulations and experimental data provided by Yamamoto *et al.* (2007),^96^ internal numbering up was taken for upscaling the plasma-based CO production process, as a small-sized reactor is required to obtain high CO_2_ conversion. A similar strategy was recently implemented in a GAP prototype, where a unified reactor body contained five *ca*. 1 kW reactor nodes operating in parallel.^100^ It was observed that the performance of each reactor node was not adversely affected by the operation of adjacent nodes. In this context, for our proposed plant, a total of 3153 reactors in parallel would meet the CO production demand.

Although the operating principles of electrolysis units for CO_2_ conversion are different from plasma reactors, the electrolysis field faces similar upscaling challenges that have gained prominence in recent research.^57,101,102^ The complexity of electrolysis systems and significant capital investment have limited the study of continuous large-scale implementation.^103^ This is relevant to address because scaled-up electrolysers often exhibit different mass transfer and reaction kinetics from their lab-scale counterparts, leading to performance disparities.^101–103^ As the cell surface area expands, ensuring efficient mass and charge transport across the larger surface becomes challenging, potentially causing uneven reactant distribution, increased ohmic losses, and limited catalyst material utilisation.^86,101,104^ Such challenges hinder the broader adoption of CO2R, primarily due to diminished current density and FE.^86^ To enhance mass and charge transport efficiency, strategies encompass configurations like H- and microfluidic-type electrolysers, optimized flow channel design, GDE, and the integration of ionic liquids-based electrolytes to mitigate solubility issues.^101–103^ Among these strategies, the implementation of GDE in upscaling lab-scale experiments has shown promise across various studies, although these studies have concluded that the high performance of the upscaled GDE-based device is not as good as its smaller counterpart.^86,103–107^ GDE-based electrolysis still holds considerable upscaling potential. Optimized performance can be achieved by selecting the right design and operational adjustments, particularly in addressing perspiration or flooding phenomena.^71,104,107^ Notably, Blake *et al.* (2023) suggested that circumventing upscaling challenges might be more preferable than prevention, thus alternative parallel geometries can be employed to maintain performance.^104^

Building on this perspective, and recognising that each cell stack necessitates its own cathode, GDE, membrane, and anode—as illustrated in the multi-stack electrolysis unit by Endrődi *et al.* (2019)^74^—this approach aligns with an external numbering-up strategy. In this context, multiple smaller cell stacks can share components that are more technically feasible and cost-effectively scalable than individual cell stacks, such as power supplies, instrumentation, and gas separation systems, as observed in analogous technologies like water electrolysis.^108,109^ Consequently, the proposed system will need a total of 61 705 cell stacks in parallel to satisfy CO production.

### 2.2 Techno-economic and energy analysis

The unitary cost of production (UCOP) per tonne of CO by the plasma- and electrolysis-based plants was estimated by considering the annual operating expenditure (Opex) and the annualized capital cost (ACC).^110^ The ACC was obtained by calculating the annual capital charge ratio (ACCR) with a 10% interest rate (*i*) and a 20-year useful life (*n*), eqn (1)–(3).1
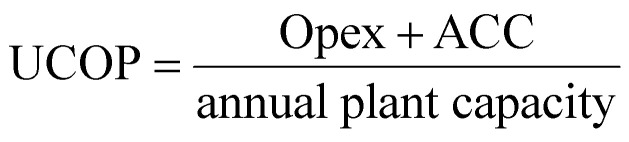

2 ACC = ACCR × Capex

3
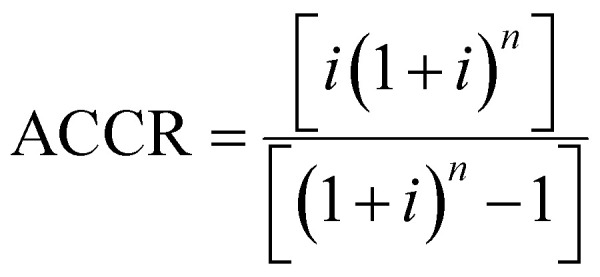


Opex includes both annual variable and fixed operating costs. The fixed operating costs, such as maintenance, supervision, and plant overheads, were estimated using a factorial approach in relation to labour costs and fixed capital expenditure (Capex). Capex corresponds to the purchase cost of equipment and direct and indirect costs of installation. The direct costs of installing the equipment, along with those of instrumentation, piping, electrical systems, buildings, site development, and indirect costs, such as engineering and supervision, construction, contractor fees, and contingency charges, were calculated using a factorial approach based on the purchase cost of equipment.^111^ The considered ratio factors were derived from the work of Anastasopoulou *et al.* (2020), supported by modelling in Aspen Plus.^112^ Due to the simplicity of these all-electric plants, the installation and maintenance costs were significantly lower than those for traditional chemical plants. For instance, the costs of piping and instrumentation, which typically constitute 66% and 18% of the equipment purchase cost, were estimated to be about 10% and 8%, respectively. The utilised factors are detailed in Table S1 of the ESI.†

Among other considerations, the working capital was assumed to be recovered at the end of the project, and therefore, not included in the Capex value.^111^ The analysed plants would produce 34 000 tonnes CO per year, considering an average of 8160 productive hours per year (*i.e.*, 340 days per year). For this production volume, the different figures of reference for equipment costs, catalyst costs, and the number of workers were scaled up using eqn (4):4
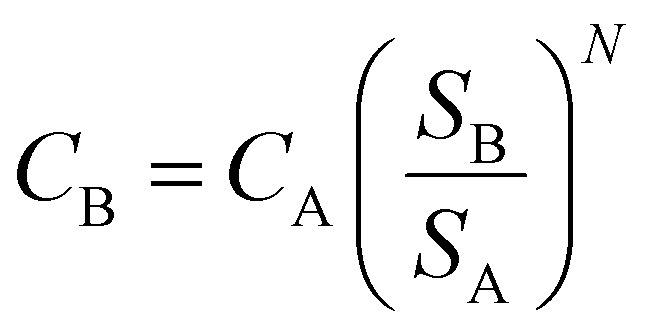


Here, *C*_B_ represents the scaled-up cost of the equipment to a given size, *C*_A_ is the known cost of the equipment with corresponding capacity *S*_A_, while *S*_B_ is the given capacity of the equipment to be scaled up, and *N* is the size exponent or ‘scaling factor’. These scaling factors are specific for each type of process or equipment and are typically lower than 1.0, which have been listed and updated by several authors.^113–116^ The rule of thumb when *N* is unknown is to use an average value of 0.6.^117^ This approach is referred to as ‘the Rule of Six-tenths,’ originally attributed to Williams Jr (1947).^118^

Before calculating the scaled-up cost (*C*_B_), the referenced known costs (*C*_A_) reported for a specific year were adjusted to US$2020 prices according to inflation rates, based on the Chemical Engineering Plant Cost Indices (CEPCI).^119^ A currency rate of 1.14 € per $ was assumed.

The workforce was estimated using a scaling factor of 0.25 based on the two operators per shift required for a similar 20 000 tonne CO per year plant^6^ and considering 4.5 operators to cover a position that needs one operator at a given time.^111,120^ This is estimated by considering that an operator works five eight-hour shifts per week for 49 weeks in a year (after subtracting sick leaves and holidays) and that a plant requires operators every day of the year, including maintenance and shutdown periods. The assumed total labour cost per operator in the chemical industry was $81.200, based on the average labour cost for high-income European countries (EU-15) in 2020.^121^

The cost of the PSA system was estimated by scaling up a 1000 m^3^ h^−1^ unit with a cost of €1.75 million in 2015^7,62,85^ and using a scaling factor of 0.67.^85^

For plasma-based systems at the lab scale, the power supply unit is typically the most expensive component. This is due to the flexibility of these power units, which can be employed with different types of plasmas, unlike the units designed for pilot or industrial scale applications that can be optimized for a specific condition. Based on colleagues' experience with a GAP upscaled prototype under the BluePlasma project^100^ and an ongoing project expected to annually process 30 000 tonnes of CO_2_ at an industrial facility in Antwerp, Belgium, by D-CRBN,^122^ we estimated a cost of 1085 € per kW (plasma power) for both the power unit and reactor body. The GAP prototype, with a yearly capacity of 6.7 tonnes of CO_2_ and a plasma power of 5.6 kW, had an approximate cost of 3000 € per kW.^100^ Since this total cost included some additional services and reworking, the actual cost of the uninstalled power unit and reactor body together was 2400 € per kW. According to the manufacturer, a similar cost can apply to plants processing up to 1000 tonnes of CO_2_ per year. For capacities beyond this range, the manufacturer suggested applying a scaling factor of 0.9.

The cost of the carbon bed amounted to less than 5% of the total expense of the entire lab-scale plasma power supply-reactor setup. However, since the lab-scale setup was overpriced due to its tailored construction, we assumed an incremental cost of 15% for the carbon bed. This results in a plasma-based CO_2_ conversion system costing 1248 € per kW (*i.e.*, 1422 $ per kW).

For CO_2_ electrolysers, an average cost of $10 000 per m^2^ was adopted. This figure falls within the range of $5000–$15 000 per m^2^ as utilized by Kibria *et al.* (2019).^60^ Moreover, it aligns with the midpoint between $920 per m^2^ employed by Jouny *et al.* (2018) and (2019),^7,59^ based on the US Department of Energy H2A model for water electrolysis,^123^ and $20 000 per m^2^ defined by Ramdin *et al.* (2021),^64^ who stated this cost lies between the SOEC and chlor-alkali electrolysers. These figures were already considered for industrial-scale plants producing around 100 CO tonne per day; therefore, no scaling factors were needed.

In relation to Opex, we assumed an average levelised cost of electricity (LCOE) of $0.03 per kW h from onshore wind energy for both CO_2_ conversion plants, based on Fasihi *et al.* (2021)^124^ and (2024),^125^ for Northern Europe in 2030. This cost has the potential to be halved by 2050. The price of CO_2_ feedstock was assumed to be $40 per tonne, as it represents the average of the range of prices utilized in previous studies for CO2R. Pure CO_2_ was utilised. Previous work has demonstrated that certain impurities such as N_2_ can possibly enhance the conversion, while H_2_O can decrease the conversion, although typically large fractions were considered.^126–129^ One advantage of the GAP reactor is that the plasma does not rely on a catalyst surface for its performance, hence, there is no risk of catalyst poisoning. However, the specific source of CO_2_ and possible impurities from the capture process were not considered in this study. The cost for the solid carbon source (Charcoal, Activated, Norit®) for the plasma reactor was $450 per tonne.^130^ The deionised water consumed in the anodic reaction in electrolysers costs $14 per m^3^.^131^ Moreover, we have included the additional operating cost to cover the replenishment of catalysts, which amounts to 2.5% of the capital cost of the electrolyser system.^7,56,62^ These fixed operating costs would be equivalent to the cost of stack renovation due to degradation. For example, stack renovation is usually performed every 7 years for polymer electrolyte membrane (PEM) water electrolysers.^132^ Since this periodic investment is considered a maintenance activity, it has been included as a fixed Opex.

As part of a sensitivity analysis, we also estimated the CO production costs by varying the prices of electricity, CO_2_ feed, and plasma or electrolyser modules, along with the main intrinsic parameters for each technology.

We also analysed the total energy per tonne of CO as an indicator of the efficiency of the plants and compared them to a conventional production method, such as partial combustion of heavy fuel oil. Due to the varied energy types and their distinct properties, we also used the life-cycle-based methods cumulative energy demand (CED),^133^ considering the lower heating values (LHV) of energy carriers, and the cumulative exergy demand (CExD)^134^ to account for primary energy expenses for the feedstocks production and facilities construction.

## 3 Results and discussion

### 3.1 Cost of production

**Fig. 4:**
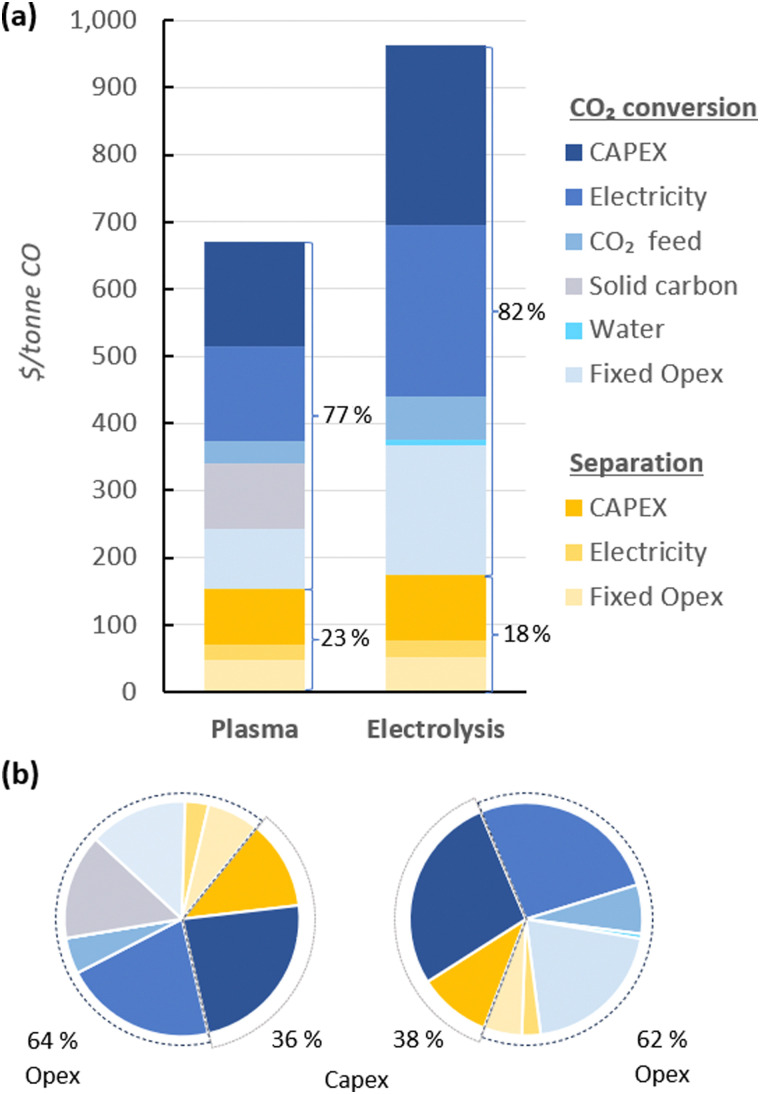
Production costs of CO *via* plasma- and electrolysis-based plants. Chart (a) represents the costs aggregated by CO_2_ conversion and separation sections (blue and yellow, respectively), and (b) represents the same contributions, with distinction between Capex and Opex (solid and dashed circle lines, respectively) to the total production cost. The underlying data for these charts is detailed in Table S5 of the ESI.†

In Fig. 4, the UCOP of CO *via* the plasma- and electrolysis-based plants are indicated, as well as the individual contributions. The total production costs are 671 and 962 $ per tonne, for the plasma- and electrolysis-based plants, respectively. These results demonstrate that all-electric plants using renewable energy could be competitive compared to the specialty price of CO transported in 40–100 L gas cylinders to the customer facilities, which could cost around 3000 $ per tonne.^6^

In both alternative plants, the costs associated with the CO_2_ conversion section are dominant, particularly in the electrolysis-based plant, where this section accounts for 82% of the UCOP of CO. On the other hand, the separation section, which was assumed to have the same capital and operational costs per volume of gas treated in both plants, required approximately 14% more resources in the electrolysis-based plant—specifically, 173 $ per tonne CO compared to 152 $ per tonne CO in the plasma-based plant, as depicted by the yellow bars in Fig. 4. This is important to highlight because it was expected that the lower CO_2_ conversion of the plasma reactor would result in a requirement for a larger PSA system and higher energy consumption due to the higher output of unreacted CO_2_ per unit of CO to be separated, but it turns out to be not the case, as explained in next paragraph.

As shown in Fig. 2, the output gas of the plasma reactor was 13 114 m^3^ h^−1^, compared to the 15 105 m^3^ h^−1^ output gas from the electrolyser (Fig. 3). However, the key to this lower separation requirement in the plasma-based plant was the incorporation of the carbon bed, which helped generate additional CO from O_2_. Consequently, less CO_2_ input was required to obtain the 4.17 tonne CO per h needed at the input of the PSA system to meet the set production capacity. The carbon bed contributed to halving the feedstock requirement from 6.81 to 3.48 tonnes of CO_2_ per h, although it also required adding 0.9 tonnes of C per h, which is ten times more expensive than CO_2_ per tonne. This means that the CO_2_ and charcoal together cost double (130.8 $ per tonne CO) compared to the cost of CO_2_ and water for the electrolysis-based plant (71.9 $ per tonne CO). However, despite these higher costs of feedstocks for the plasma reactor, the reduced CO_2_ feed also decreased the energy requirements and the power unit size, thereby reducing the Capex and electricity consumption in this plant. This is relevant because the electricity cost for the CO_2_ conversion represents about 21% and 27% of the total costs in plasma and electrolyser plants, respectively. However, in absolute terms, the electricity consumed in the electrolyser is about 82% higher than in the plasma reactor per unit of CO produced.

Additionally, another significant portion of the costs in the CO_2_ conversion section of the electrolysis-based plant is attributed to Capex, which accounted for 34% of the costs of the section (represented by the darkest of the blue bars), while the remaining 66% was related to Opex. It is important to note, however, that 40% of the total Opex corresponds to fixed Opex, which is primarily linked to Capex. This is because fixed Opex includes, besides personnel costs, other items calculated as fractions of the Capex. These items encompass maintenance, catalysts (*e.g.*, stack renovation), insurance, and property taxes, contributing to 89% of the total fixed Opex. Therefore, if the cost of the electrolyser decreases, fixed Opex would also substantially decline. This finding also underscores that one of the primary strategies for reducing the UCOP must focus on the electrolyser cost, which remains uncertain, as indicated in the revised literature.

The Capex : Opex ratios in Fig. 4(b) suggest that for both the plasma-based and, to a greater extent, the electrolysis-based plant, the assumed equipment costs are high because commercial-scale plants typically exhibit a ratio of 30 : 70. In this context, the assumption of $10 000 per m^2^ for an uninstalled electrolyser might contribute to the high UCOP, especially when compared to previous studies that estimated costs around $285 per tonne of CO by assuming a cost of $920 per m^2^ for an installed electrolyser.^59^ Furthermore, the authors assumed a 50% SPC, as well as lower electricity costs, lower cell voltage, and higher current density compared to the parameters considered in our study.

Regarding Capex in the plasma-based plant, not many techno-economic studies for this type of technology have been published to compare the quoted costs for the reactor and power supply unit. This uncertainty is further exacerbated by the existence of various types of plasmas, each having very different costs. For instance, Kaufmann *et al.* (2023)^79^ and Lamberts-Van Assche *et al.* (2022)^5^ estimated a cost of €1300 per kW for a DBD plasma reactor, with power requirements of 3000 kW and 144 kW, respectively. However, it is important to note that our GAP plasma reactor system required a power of 15 645 kW, and its cost was derived from an actual operating system, leading us to believe this number is reliable. This can be attributed to the fact that while such systems typically scale up almost linearly, suppliers can optimize production and provide discounts for bulk purchases. This optimization can reduce the linear scaling factor (1.0) to values close to 0.8, a trend similar to that observed for water electrolysers.^135,136^

Overall, the values of different parameters used for the base cases must be varied in a sensitivity analysis, especially those with a significant impact on the total costs or those that have shown high dispersion according to the values used in previous studies and are sensitive to market fluctuations. These parameters primarily include extrinsic factors, such as the cost of electricity, CO_2_ input, charcoal input, and the purchase cost of CO_2_ conversion equipment (plasma and electrolysis units).

Furthermore, the most relevant intrinsic parameters are those that directly contribute to the energy cost in both plants, such as cell voltage and plasma power. Inversely related parameters, like the SPC, also play a crucial role. When these values are lower, they increase the amount of CO_2_ required as input, consequently demanding more energy for the conversion and the separation, as well as bigger equipment. The effect is the opposite when SPC values are higher. However, it is important to consider that usually, to obtain higher SPC values, the gas flow rate should be reduced to increase the residence time, which also increases the specific energy input (MJ m^−3^). In other words, high SPC values help reduce the quantity of CO_2_ required and the size of the equipment, but they also increase the power required per unit of CO_2_ converted.

Another intrinsic parameter affecting Capex is current density in electrolysers. Higher current densities reduce the required cell area, but it is crucial to note that it also leads to an increase in cell voltage. Therefore, this parameter must be adjusted when there is a change in the energy consumption of the electrolysis cell. FE is also a relevant parameter for energy consumption. However, recent studies have consistently shown this parameter to remain between 80% and 90%. Therefore, it is unnecessary to include it in a sensitivity analysis.

### 3.2 Sensitivity analysis

**Fig. 5:**
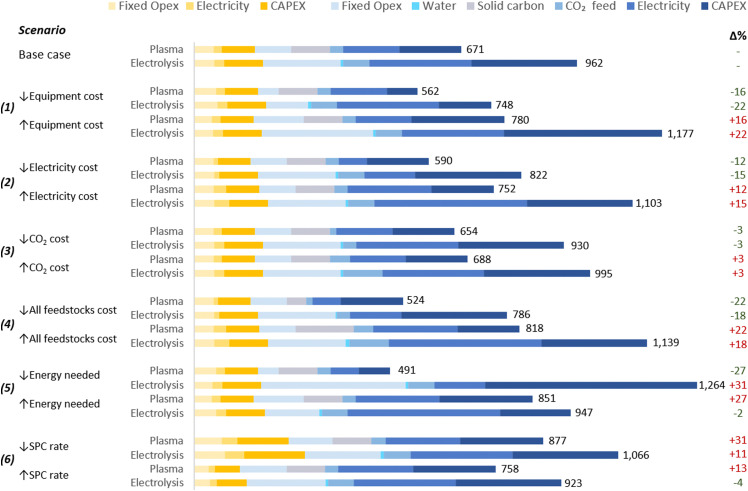
Scenarios for sensitivity analysis of CO production costs. Values on the right of the bars represent the UCOP [$ per tonne CO], and numbers in green and red in the right represent the percentage variation of the UCOP with respect to the base cases. Legends’ colours are the same as those in Fig. 4. The underlying data for this chart is detailed in Table S5 of the ESI.†

The proposed scenarios involved a ±50% variation in the following extrinsic parameters: (1) the cost of the plasma system and electrolyser; (2) electricity cost; (3) CO_2_ cost; and (4) all feedstock costs. Moreover, two additional scenarios for the analysis of intrinsic parameters were considered: (5) energy costs of CO_2_ conversion; and (6) SPC values. The scenarios are detailed in Table 3 and the results in Fig. 5.

**Table 3:** Parameter values for the base case and sensitivity analysis scenarios for plasma- and electrolysis-based CO production. In scenarios 1–3 and 5–6, one parameter was always enhanced/reduced by 50%, as indicated by the up/down arrows. In scenario 4, several parameters (*i.e.*, all feedstock costs) were changed simultaneously. In scenario 6, changes in other parameters occurred indirectly following the variation of the SPC

Parameter	Unit	Base case			(1)			(2)			(3)			(4)			(5)^b^			(6)^d^	
		Plasma	Electrol.		Plasma	Electrol.		Plasma	Electrol.		Plasma	Electrol.		Plasma	Electrol.		Plasma	Electrol.		Plasma	Electrol.
Conversion equipment cost	$ per kW^a^	1,422	10 000	↓	711	5000		1422	10 000		1422	10 000		1422	10 000		1422	10 000		1422	10 000
				↑	2133	15 000															
Electricity cost	$ per kW h	0.03	0.03		0.03	0.03	↓	0.015	0.015		0.03	0.03	↓	0.015	0.015		0.03	0.03		0.03	0.03
							↑	0.045	0.045				↑	0.045	0.045						
CO_2_ feed cost	$ per tonne	40	40		40	40		40	40	↓	20	20	↓	20	20		40	40		40	40
										↑	60	60	↑	60	60						
Water cost	$ per m^3^		14			14			14			14	↓		7			14			14
													↑		21						
Solid carbon cost	$ per tonne	450			450			450			450		↓	225			450			450	
													↑	675							
Conversion energy cost c	kW h per kg CO	4.69	8.53		4.69	8.53		4.69	8.53		4.69	8.53		4.69	8.53	↓	2.35	4.27		6.26	8.53
																↑	7.04	12.80		6.26	
SPC	%	16	25		16	25		16	25		16	25		16	25		16	25	↓	8	12.5
																			↑	24	37.5
Separation energy cost	kW h per kg CO	0.724	0.834		0.724	0.834		0.724	0.834		0.724	0.834		0.724	0.834		0.724	0.834		1.343	1.628
																				0.517	0.570

a The cost unit for electrolysers is $ per m^2^.

b The plasma reactors require a power of 15 645 kW, based on the 835 W needed for a 10 000 cm^3^ min^−1^ reactor in the base case—a power that was varied by ±50% in scenario 5. For electrolysers, a total power of 28 436 kW and cell area of 3791 m^2^ are required in the base case. In scenario 5, the cell voltage (3.0 V) was varied by ±50%, along with a current density (250 mA cm^−2^) variation to the same extent. This resulted in not only a proportional direct variation in the required power but also a proportional inverse variation in the cell area needed.

c The energy required considers an 80% plug-to-equipment efficiency.

d In scenario 6 for the plasma-based plant, the SPC was varied by ±50%, while the CO_2_ flow rate was adjusted to the same extent but in the opposite direction. This led to a direct proportional change in energy consumption with the SPC and an inverse change with the CO_2_ flow rate, resulting in a combined increase in the conversion energy cost by +33% in both cases. For the electrolysis- and plasma-based plants, the variation in the SPC results in an inverse variation in the energy cost required for CO separation.

These hypothetical variations were determined due to the challenge of predicting exact changes in parameters, given the early stages of the analysed technologies. Further engineering developments could potentially reduce CO_2_ conversion equipment costs, while market fluctuations, such as changes in material prices, might lead to increases. Similarly, efforts to enhance carbon capture technologies might lower CO_2_ costs, yet rising demand from other industries could inflate prices.

Electricity costs are more predictable, albeit subject to variations between countries due to resource availability, market dynamics, and sociopolitical conflicts. The projected reduction in LCOE, estimated at $0.015 per kW h by 2050, is expected in several regions for wind and solar photovoltaic (PV) farms.^124,125^ Additionally, this low cost could be achieved before 2030 in virtually all countries located below the parallel 45°N, particularly through the use of solar PV.^124,125^

As expected, in scenarios where changes in selected parameters (scenarios 1–4) did not affect other parameters, the impact of each respective change depended on the contribution of the specific parameter to the total UCOP of CO. For example, a ±50% change in the cost of the CO_2_ conversion equipment (scenario 1) resulted in a ±16% and ±22% change in the UCOP of CO for plasma-based and electrolysis-based plants, respectively. The impact was higher on the electrolysis-based plant, mainly due to the significant contribution of Capex and fixed costs of the CO_2_ conversion section in the base case. In this scenario, with a lower cost ($5000 per m^2^) for the electrolyser, the Capex:Opex ratio of the plant would be closer to 30 : 70 and achieve its lowest UCOP ($748 per tonne CO).

In scenario (2), a ±50% change in electricity costs led to a ±12% and ±15% variation in the UCOP for the plasma- and electrolysis-based plants, respectively. This corresponds to fluctuations of ±$81 and ±$140 per tonne of CO.

In scenario (3), the variation in the CO_2_ feed costs only affected the UCOP by 3% in both plants. This was because, in the plasma-based plant, electricity and solid carbon costs are more relevant, while in the electrolyser-based plant, electricity and equipment costs are more significant.

On the other hand, more notable impacts of ±22% were obtained in scenario (4), where the relatively significant contribution of feedstocks, including solid carbon, CO_2_, and electricity, changed from 44% in the base case to 28% and 54% when feedstock costs were reduced and increased, respectively.

In scenario (5), variations in plasma power resulted in a ±27% change in UCOP not only due to the different electricity requirements but also to the different size of required power supply unit-reactor system.

In contrast to scenarios where changes in parameter costs affected the UCOP of both plants in the same direction, the results were unpredictable when the variation of one parameter implied a comparable variation in another parameter. For the electrolysis-based plant, when the cell voltage was reduced in scenario (5), instead of decreasing the UCOP, it resulted in a 31% increase in UCOP, the highest among all scenarios ($1264 per tonne CO). This occurred because the current density was reduced to a similar extent as the cell voltage, leading to a dramatic increase in the Capex and fixed costs of the CO_2_ conversion section. This effect was also influenced by the relatively high cost of the electrolysis cell considered in the base case. If we assumed a reduced electrolyser cost of $5000 per m^2^ in this scenario, the impact of the reduced current density would have been less significant than the benefit of the reduced cell voltage, resulting in a UCOP of $834 per tonne CO, hence 13% lower than in the base case.

In scenario (6), when the SPC was reduced by 50%, the CO_2_ input flow rate increased to a similar extent, while a constant plasma power was assumed. In this context, a higher CO_2_ input flow rate leads to lower specific energy input for plasma-based CO_2_ conversion. However, since halving the SPC implies the duplication of the CO_2_ feed to the reactor due to the high volume of recycled CO_2_, the total power required in the reactor increases by a third, resulting in higher electricity costs and Capex. Therefore, in addition to the higher separation costs and the higher CO_2_ feed required due to doubled CO_2_ losses in the separation system, which affects both kinds of plants, the UCOP of CO from plasma-based plants is more affected by variations in SPC. When the SPC was reduced in both plants, the UCOP of CO increased by 31% and 11% for the plasma- and electrolysis-based plants, respectively.

When the SPC was increased, reduced costs were expected due to halving the CO_2_ feed to the reactor and the gas to be treated in the separation system. However, this CO_2_ input reduction increased the specific energy input in the plasma reactor, leading to higher energy consumption and Capex in the CO_2_ conversion section. This resulted in a higher UCOP by 13%.

In contrast, changes in SCP had a low impact on the UCOP of CO in electrolysis-based plants, affecting only the separation section. This is because in CO_2_ conversion *via* electrolysis, the energy required for the reaction is not calculated based on the CO_2_ passed but on the cell voltage and the current, the latter determined by the quantity of CO converted per hour and the FE. Therefore, in scenarios with constant cell voltage, CO output rate, and FE, any change in the SPC does not affect the energy efficiency of the CO_2_ conversion process. Due to the limited relevance of this parameter in the efficiency of the conversion, this might be one of the reasons why SPC values are not frequently reported in publications on CO2R.

The above analyses for scenarios (5) and (6) demonstrate that conducting a TEA based on intrinsic parameters from different studies or randomly modifying them for sensitivity analyses can lead to unreliable results. This is because, as the example for the case of electrolysers, optimal parameters such as high current density and low cell voltages are not typically achieved in the same experimental setting. Furthermore, assuming that all other parameters remain constant is not recommended when adjusting SPC and CO_2_ flow rates. For example, increasing the CO_2_ flow rate can also impact the FE. This is because alterations in CO_2_ flow rates may result in changes in reaction kinetics, electrode performance, overpotential, cell temperature, and catalytic activity, which in turn could lead to changes in FE, but the extent and direction of that change will depend on these factors and may require experimental validation.

This is why we considered it essential to perform our TEA based on intrinsic parameters obtained in a single experimental setup for each type of plant. This approach increases the consistency and transparency of the study and reduces uncertainties in the results. For this reason, results from scenarios (1)–(4), where only extrinsic parameters were modified, can be considered more reliable than results from scenarios (5) and (6).

Nevertheless, despite the reliability of these results, given the significant impact of certain parameters, such as electricity and equipment costs, coupled with their high uncertainty or variability due to external factors, it is worthwhile to individually analyse their impact on the UCOP beyond the ±50% variations.

### 3.3 Parameter-specific analysis

Although the LCOE for users owning renewable energy plants has decreased in recent decades,^124,125^ market dynamics can affect these costs, particularly for electricity purchased through third-party suppliers. Regarding equipment costs, the concern lies in the uncertainty surrounding the actual cost of CO_2_ conversion systems, especially for CO_2_ electrolysers, where costs between $920 and $20 000 per m^2^ have been reported.^7,59,64^

**Fig. 6:**
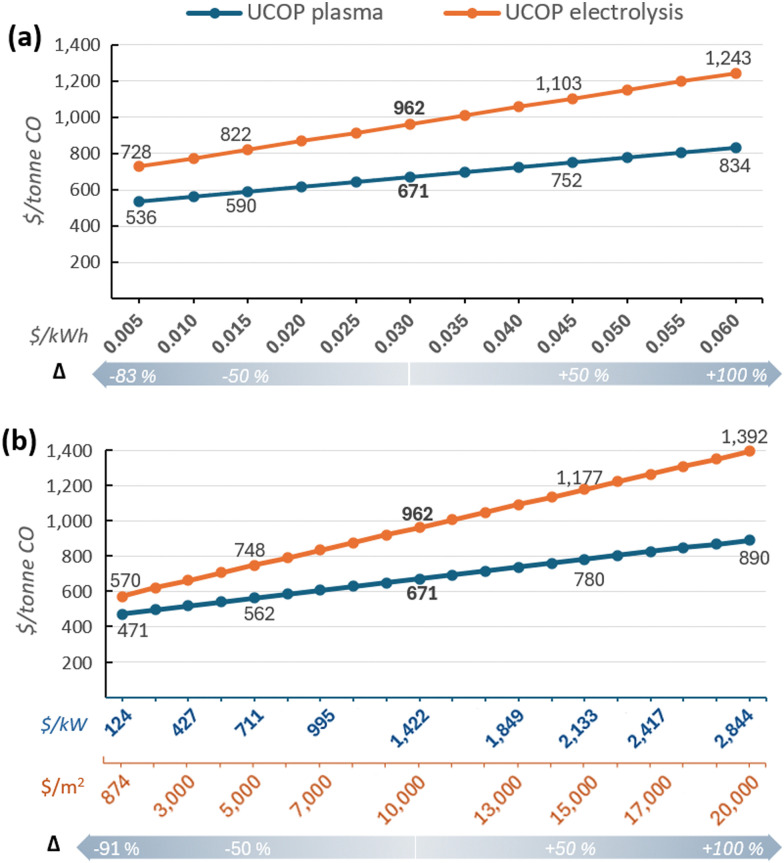
Sensitivity analysis: (a) Electricity cost *vs.* UCOP of CO; (b) CO_2_ conversion equipment cost *vs.* UCOP of CO. Values on the *X*-axis in blue represent the cost of the plasma system ($ per kW), while values in orange represent the cost of the electrolyser ($ per m^2^). The underlying data for these charts are detailed in Tables S6 and S7 of the ESI.†

In Fig. 6(a), the electricity cost was varied from $0.005 to $0.06 per kWh, representing a variation between −83% and +100% compared to the base case. Concerning equipment cost in Fig. 6(b), it was varied between −91% to 100%. The lowest cost for CO_2_ electrolysers, reported as $920 per m^2^,^7,59^ required updating because it was based on a cost of $250 per kW (US$2010), which also included a 20% extra charge for installation. Therefore, after removing the installation cost and updating to US$2020, the uninstalled cost of an electrolyser would be $874 per m^2^.

In the scenario with an electricity cost of $0.06 per kW h, the UCOP of CO in plasma- and electrolyser-based plants would be $834 and $1243 per tonne, respectively, increasing by 24% and 29% compared to the base cases. The impact in electrolyser-based plants was slightly higher, as observed in the slopes of the lines in Fig. 6(a).

The variation in equipment cost in Fig. 6(b) had a greater impact than electricity cost in both plants, although the impact was more pronounced in the electrolyser-based plant. In this plant, the UCOP of CO would range between $570 and $1392 per tonne, representing a variation of over ±40% compared to the base case under scenarios of minimum and maximum electrolyser cost. For plasma-based plants, the UCOP of CO would vary by approximately ±30%.

In both analyses presented in Fig. 6, variations in the cost of electricity and CO_2_ conversion equipment had a lower impact in plasma-based plants due to other parameters with significant participation in the UCOP of CO, such as the solid carbon input.

**Fig. 7:**
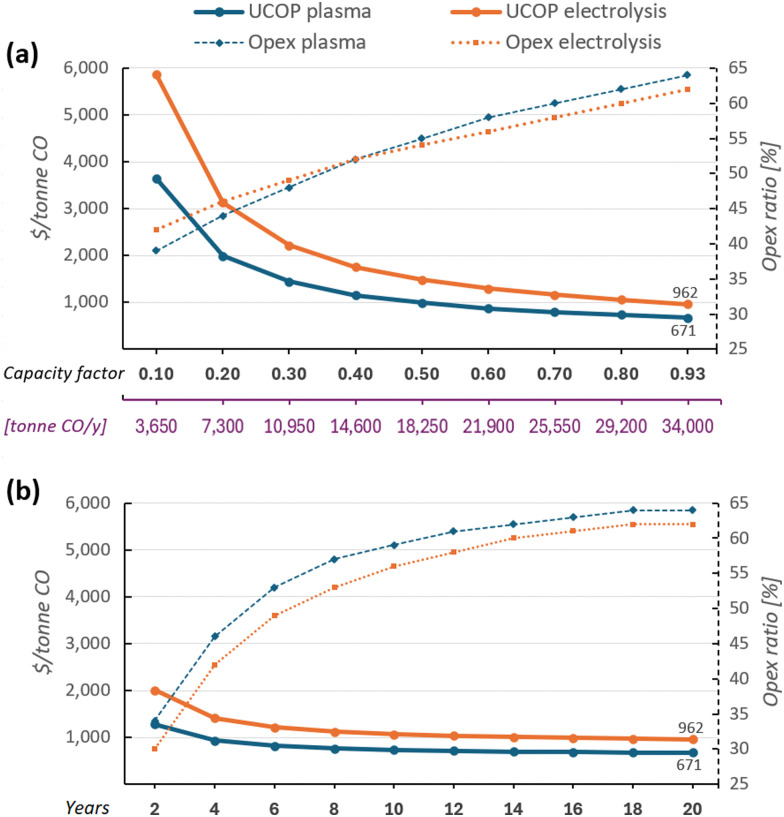
Sensitivity analysis: (a) Capacity factors *vs.* UCOP of CO; (b) CO_2_ conversion equipment lifespan *vs.* UCOP of CO. Solid lines represent UCOP of CO (left *Y*-axis), while dashed lines represent the ratio of Opex to UCOP of CO (right *Y*-axis). The underlying data for these charts are detailed in Tables S8 and S9 of the ESI.†

It is important to note that in the short term, increases rather than reductions in the analysed parameters are more likely, especially in electricity costs. Although self-managed renewable energy plants are less affected by market or sociopolitical events, the intermittency of energy sources or meteorological events may require the use of more expensive conventional energy sources as backups to maintain the assumed capacity of 0.93 (*i.e.*, 8160 h year^−1^). However, given the flexibility of these plants and the ability to operate on demand, backups may not be necessary. Therefore, a sensitivity analysis considering lower plant capacity factors is presented in Fig. 7(a).

Another source of uncertainty affecting the UCOP of CO is the plant lifespan. The 20-year useful life, as considered in most previous studies,^5,7,64,137–139^ was assumed in this analysis to maintain comparability of the results. However, given the technological immaturity of both technologies, equipment durability remains unknown. For instance, while no stack renovation due to catalysis degradation is necessary for GAP reactors, as experienced in electrolysers,^132^ some plasma reactors may eventually suffer from electrode damage and wall erosion. Therefore, a sensitivity analysis considering different lifespans is presented in Fig. 7(b).

Reducing capacity factors had the highest impact on UCOP in both plants. Cutting production by almost half, increases the UCOP of CO to around 1000 and 1500 in plasma- and electrolysis-based plants, respectively; see Fig. 7(a). It is also observed that lower annual production results in a reduced Opex ratio. However, considering that Opex includes fixed costs related to maintenance of equipment, if only 10% of the annual installed capacity were produced, Opex would still have a relevant participation of the UCOP of 39% and 42% in plasma- and electrolysis-based plants, respectively. A similar trend in Opex ratio occurred when CO_2_ conversion equipment required earlier replacement, Fig. 7(b). However, replacing the main systems had a less significant impact on UCOP compared to capacity factors. For instance, if the CO_2_ conversion equipment lasts 10 years, UCOP per tonne of CO in plasma- and electrolysis-based plants would be $731 and $1065, respectively, representing increases of only 9% and 11%.

Apart from capacity factors, it has been observed that varying individual factors does not result in extreme changes in UCOP of CO, as no single parameter is responsible for the majority of the total UCOP. The plasma-based plant benefits from a favourable distribution of cost shares due to the use of the carbon source. Despite the significant participation of Opex, it also helps reduce CO_2_ input, electricity consumption, and Capex. In this context, considering scenarios with various changes in extrinsic parameters, analysing an optimistic scenario could reveal the competitiveness of these CO_2_ conversion technologies in the future.

### 3.4 Optimal scenario

**Fig. 8:**
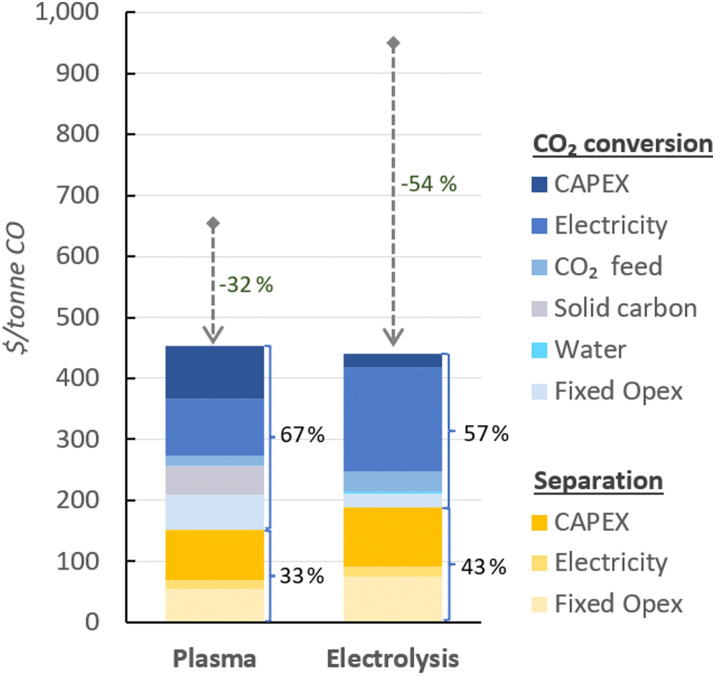
Optimal scenario for alternative CO production plants. The values next to the bars indicate the contribution of CO_2_ conversion and separation sections to the UCOP, while the values on top of the bars represent the percentage variation relative to the UCOP of the base cases.

For the most optimal scenario, we have considered minimum costs for electricity ($0.02 per kW h), CO_2_ input ($20 per tonne), charcoal ($225 per tonne), and water ($7 per m^3^). The cost of the plasma reactor setup was set at 787 $ per kW, including the 15% increment for the carbon bed, based on the estimated cost of the power unit-reactor body of 600 € per kW for future plasma-based plants in industrial environments.^100^ This represents a reduction of 45% compared to the equipment cost in the base case. This reduction may be the highest achievable because, given the simplicity of design and the use of common materials, future research and engineering efforts might not yield higher cost reductions than those achievable through economies of scale. For the electrolyser cost, we assumed the lowest value of $874 per m^2^. The results of this scenario are presented in Fig. 8.

In this optimal scenario featuring low-cost feedstocks and CO_2_ conversion equipment, the UCOP would be approximately $454 and $441 per tonne of CO for the plasma- and electrolysis-based plants, respectively. The significant reduction in UCOP for the electrolysis-based plant is primarily attributed to the low electrolyser cost, which accounted for only 5% of the UCOP, while the separation equipment represented 22%. However, it is important to note that this result should be viewed as a reference for the potential minimum cost achievable in the distant future. This is because the assumed electrolyser cost is unlikely to align with current CO2R equipment designs. Traditionally, alkaline electrolysers have been more cost-effective than other water electrolysers, such as PEM electrolysers. This cost advantage is due to their use of lower-cost materials like nickel-based electrodes and simpler designs. In contrast, PEM electrolysers typically require expensive platinum-based catalysts, which is also the case for CO_2_ electrolysers, which typically rely on expensive catalysts, including silver- and titanium-based materials. Therefore, achieving low prices for CO_2_ electrolysers, as commercially mature alkaline electrolysers, may not be expected in the near future.

On the other hand, the estimated cost of CO production *via* plasma reactors may be more plausible due to their simpler design and the absence of expensive catalysts. This simplicity results in less complex manufacturing and opens the possibility for a broader range of manufacturers to supply equipment at more reasonable prices.

### 3.5 Energy cost analysis

**Fig. 9:**
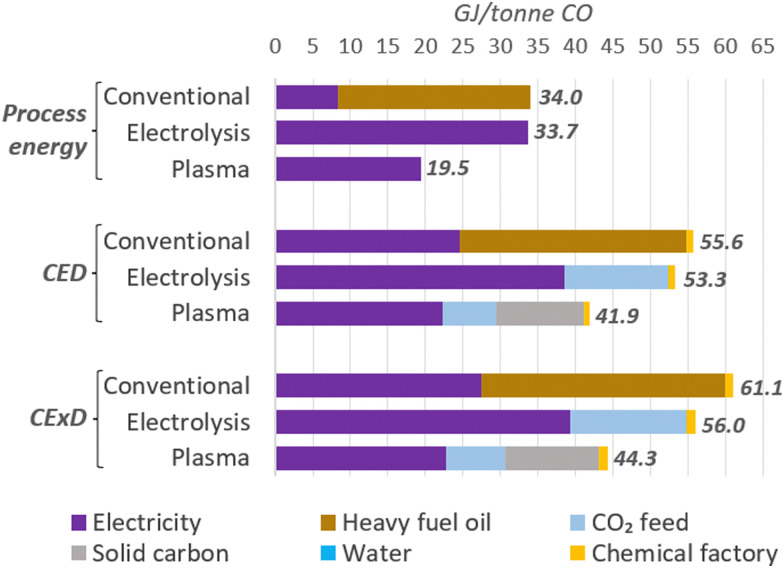
Energy costs for conventional and electrolysis- and plasma-based CO production methods (base cases).

In terms of energy cost, the conventional method for CO production, which involves the partial combustion of heavy fuel oil, utilises 2.30 MW h of electricity and 660 kg of heavy fuel oil per tonne of CO.^140^ Considering the LHV of heavy fuel oil to be 39 MJ per kg, the total energy consumed in the process amounts to 34 GJ per tonne of CO. The entire electrolysis-based plant consumes 9.36 MW h per tonne of CO (*i.e.*, 8.53 + 0.834 kW h per kg CO, as depicted in Fig. 3), equivalent to 33.7 GJ per tonne of CO—matching the energy cost of the conventional method; Fig. 9. In contrast, the plasma-based method requires 5.41 MW h per tonne of CO (as shown in Fig. 2), or 19.5 GJ per tonne of CO, representing a 43% reduction in total energy cost.

In a life cycle context, considering the energy spent to produce the electricity and supplies, the results for the CED show that the conventional method accounts for 55.6 GJ per tonne CO, with 54% from heavy fuel oil and 44% from the grid electricity; see Fig. 9. The electrolysis-based method uses 53.3 GJ per tonne CO, with 72% from wind power and 26% from CO_2_ feedstock. The plasma-based method consumes 41.9 GJ per tonne CO, with contributions of 53% from wind power, 28% from charcoal, and 17% from CO_2_ feedstocks. Notably, the plasma-based method reduces CED by only 21% compared to electrolysis-based and 25% compared to the conventional method. This modest reduction stems from the energy-intensive process of producing charcoal, which requires significant heat for hardwood drying, despite being a more energy-efficient solid carbon source than charcoal derived from mineral hard coal.

Regarding CExD, the conventional, electrolysis-, and plasma-based methods account for 61.1, 56.0, and 44.3 GJ per tonne CO, respectively; see Fig. 9. Thus, the plasma-based plant reduces the demand by 21% compared to the electrolysis-based and 27% compared to the conventional method. The increased exergy demand of the conventional method arises from its reliance on heavy fuel oil, leading to significant exergy losses during combustion. Additionally, the exergy inefficiencies of grid electricity, attributable to generation, transmission, and distribution processes, especially when sourced from fossil fuels, impact the conventional method more than alternative methods utilising higher-quality local energy sources.

## 4 Conclusions

The techno-economic analysis of small-scale CO production using plasma- and electrolysis-based CO_2_ conversion indicates promising competitiveness in future industrial landscapes. Presently, electrolysis for CO2R faces high and uncertain equipment costs due to complexity and expensive catalysts, contrasting with GA plasma reactors, which are simpler and use more cost-effective materials. Notably, estimated production costs per tonne of CO in plasma-based plants stand at $671, in contrast to $962 in electrolysis-based counterparts. However, considering the high uncertainty surrounding the electrolyser costs, ranging from $874 per m^2^ to $20 000 per m^2^, the cost per tonne of CO in electrolysis-based plants may vary from $570 to $1392. Despite this uncertainty, these all-electric plants using renewable energy show competitiveness against CO transported in gas cylinders, which can cost up to $3000 per tonne. It is noteworthy that electricity costs remain significant for both alternatives but are expected to decrease in relevance due to low prices from renewable sources in the future.

Another key factor contributing to the low CO production cost for the plasma-based plant is also the incorporation of the carbon bed in the plasma reactor. This component facilitates the generation of additional CO from the O_2_ produced during the initial stage of plasma-based CO_2_ decomposition. It also enhances the SPC by converting more CO_2_ to CO *via* the reverse Boudouard reaction. Consequently, with a higher concentration of CO at the reactor outlet, less CO_2_ gas input is required in the feed. This reduces plasma power requirements, equipment size, and separation expenses.

For electrolysis-based plants, addressing the elevated cost of electrolysis cells per m^2^ and the low current densities achieved in the process (mA cm^−2^) would substantially decrease the estimated CO production cost. Nevertheless, it is important to consider that other parameters might be affected when aiming for higher current densities, such as the increase in cell voltages or the impact on FE and SPC when modifying the CO_2_ input flow.

In an optimal scenario that considers low-cost feedstocks and CO_2_ conversion equipment, CO production costs could fall below $500 per tonne for both types of plants. However, achieving such a scenario is less likely for electrolysis-based plants in the near future, due to the complexity of CO_2_ electrolysers and the use of more expensive catalysts. In contrast, CO production through plasma reactors appears more feasible, due to their simpler design, the absence of costly catalysts, and the potential for wider manufacturer participation at competitive prices.

In this context, plasma-based CO production could also compete effectively against traditional plants, especially when considering the fact that this process consumes 43% less energy than its competitors, which could position it as a competitive and environmentally friendly choice for the future.

Finally, in this study, we only compared plasma technology with low-temperature electrolysis and not with SOEC technology, as the different operational characteristics of the latter would not allow for direct comparison. Nevertheless, we acknowledge that SOEC technology is at a more advanced stage of maturity and is already commercially available.

## Author contributions

J. Osorio-Tejada: writing – original draft, visualization, investigation, conceptualization, methodology. M. Escriba-Gelonch: visualization, investigation. R. Vertongen: visualization, investigation, writing. A. Bogaerts: writing – reviewing and editing, supervision. V. Hessel: writing – reviewing and editing, supervision.

## Abbreviations

ACC: Annualised capital cost
ACCR: Annual capital charge ratio
Capex: Capital expenditure
CED: Cumulative energy demand
CEPCI: Chemical engineering plant cost indexes
CExD: Cumulative exergy demand
CO: Carbon monoxide
CO_2_: Carbon dioxide
CO2R: Carbon dioxide reduction
DBD: Dielectric barrier discharge
FE: Faradaic efficiency
GA: Gliding arc
GAP: Gliding arc plasmatron
GDE: Gas diffusion electrodes
KOH: Potassium hydroxide
LCOE: Levelised cost of electricity
LHV: Lower heating value
MCEC: Molten carbonate electrolysis cells
MW: Microwave
NPW: Novel process windows
Opex: Operating expenditure
PEM: Polymer electrolyte membrane
PSA: Pressure swing adsorption
PV: Photovoltaic
SMR: Steam methane reforming
SOEC: Solid oxide electrolysis cells
SPC: Single-pass conversion
TEA: Techno-economic analysis
UCOP: Unitary cost of production

## Conflicts of interest

There are no conflicts to declare.

## Supplementary Material

EE-017-D4EE00164H-s001
