# Supplementary material for: CO2 conversion to CO via plasma and electrolysis: a techno-economic and energy cost analysis
Source: Energy Environ Sci. 2024 May 6;17(16):5833–53. doi: 10.1039/d4ee00164h (PMC11320396; doi:10.1039/d4ee00164h)
Supplement: EE-017-D4EE00164H-s001 [file EE-017-D4EE00164H-s001.pdf]

Electronic Supplementary Material (ESI) for Energy & Environmental Science.
This journal is © The Royal Society of Chemistry 2024

Supplementary Material file for the article:

CO_2 conversion to CO via plasma and electrolysis: a techno-economic and energy cost analysis

Jose Osorio-Tejada[1,5], Marc Escriba-Gelonch[2], Rani Vertongen[3], Annemie Bogaerts[3,*], Volker Hessel[1,4,*]

[1]School of Engineering, University of Warwick, United Kingdom
[2] Department of Environment, Soil Sciences and Chemistry, University of Lleida, Spain
[3] Research Group PLASMANT, Department of Chemistry, University of Antwerp, Belgium
[4] School of Chemical Engineering, University of Adelaide, Australia
[5]Faculty of Environmental Sciences, Universidad Tecnológica de Pereira, Colombia

* Authors for correspondence: annemie.bogaerts@uantwerpen.be; volker.hessel@adelaide.edu.au

This Supplementary Material file presents the results of the techno-economic analyses for each CO production pathway in which capital and operating costs and the unitary cost of production (UCOP) are detailed. The methods, references, and assumptions for the calculation process are described in the main article.

Contents

Table S1. *Capital costs for the plasma- and electrolysis-based plant (34,000 t CO/year)*
Table S2. *Operating costs for the plasma- and electrolysis-based plant (34,000 t CO/year)*
Table S3. *Total annual costs for the plasma- and electrolysis-based plants (34,000 t CO/year) by sections*
Table S4. *Unitary cost of production (UCOP) for the plasma- and electrolysis-based plants by sections*
Table S5. *Unitary cost of production (UCOP) for the plasma- and electrolysis-based plants by sections and scenarios*
Table S6. *Sensitivity analysis: Fig. 6(a) Electricity cost vs UCOP of CO*
Table S7. *Sensitivity analysis: Fig. 6(b) CO2 conversion equipment cost vs UCOP of CO*
Table S8. *Sensitivity analysis: Fig. 7(a) Capacity factors vs UCOP of CO*
Table S9. *Sensitivity analysis: Fig. 7(b) CO2 conversion equipment lifespan vs UCOP of CO*

Table S1

Capital costs for the plasma- and electrolysis-based plants (34,000 t CO/year)

**CAPITAL COSTS**	**(US$)**		**(%)**	
	Plasma-based	***Electrolysis-based***		
**Purchase Cost of Equipment (PCE)**	**34,035,612**	**51,815,140**		
CO_2 conversion equipment	22,261,965	37,914,156		
Separation system equipment	11,773,648	13,900,985		
**Direct costs**	**58,200,897**	**88,603,890**	Direct cost factors	
Purchase Cost of Equipment (PCE)	34,035,612	51,815,140	100%	of PCE
Equipment installation	8,508,903	12,953,785	25%	of PCE
Instrumentation/controls installation	2,042,137	3,108,908	6%	of PCE
Piping installation	3,403,561	5,181,514	10%	of PCE
Electrical systems installation	3,403,561	5,181,514	10%	of PCE
Buildings	3,403,561	5,181,514	10%	of PCE
Site development	3,403,561	5,181,514	10%	of PCE
**Indirect costs**	**10,969,678**	**16,700,020**	Indirect cost factors	
Engineering and supervision	2,910,045	4,430,194	5%	of direct costs
Construction expenses	3,492,054	5,316,233	6%	of direct costs
Contractor fees	1,164,018	1,772,078	2%	of direct costs
Contingency fees	3,403,561	5,181,514	5%	of FCI
**Fixed Capital Investment (FCI)**	**69,170,575**	**105,303,909**	Direct costs + Indirect costs	

Table S2

Operating costs for the plasma- and electrolysis-based plants (34,000 t CO/year)

**OPERATING COSTS**	**Annual costs (US$)**		
	Plasma-based	***Electrolysis-based***	
**Total variable costs**	**9,973,294**	**11,997,826**	Raw materials + Utilities
**Raw materials**	**4,447,640**	**2,445,708**	
CO_2 feed	1,135,952	2,223,492	
Solid carbon feed	3,311,688	222,216	
**Utilities**	**5,525,613**	**9,552,118**	
Electricity for CO_2 conversion	4,787,251	8,701,299	
Electricity for separation section	738,363	850,820	
**Total fixed costs**	**4,724,797**	**8,349,387**	
Maintenance	1,383,412	2,106,078	2% of FCI
Electrolysis cells stack renovation	0	1,926,323	2.5% of the FCI (only electrolyser)
Operating labour	812,000	812,000	Actuals ($)
Laboratory charges	81,200	81,200	10% of operating labour
Plan overheads and general expenses	1,479,797	1,949,531	65% of labour and maintenance costs
Insurance	276,682	421,216	0.4% of FCI
Taxes	691,706	1,053,039	1% of FCI
**Total Operating costs**	**14,698,018**	**20,347,213**	Variable costs + Fixed Costs

Table S3

Total annual costs for the plasma- and electrolysis-based plants (34,000 t CO/year) by sections

	Total Annual costs (US$)					
	Plasma-based			***Electrolysis-based***		
	CO_2 conversion	Separation	TOTAL	CO_2 conversion	Separation	TOTAL
**Annualised capital costs**	**5,314,225**	**2,810,525**	**8,124,750**	**9,050,609**	**3,318,348**	**12,368,958**
**Capital costs**	**45,242,991**	**23,927,585**		**77,052,938**	**28,250,971**	
PCE	22,261,965	11,773,648		37,914,156	13,900,985	
Installation	22,981,026	12,153,937		39,138,783	14,349,986	
**Annual operating costs**	**12,325,246**	**2,372,771**	**14,698,018**	**17,773,212**	**2,574,001**	**20,347,213**
CO_2 feed	1,135,943			2,223,492		
Solid carbon feed	3,311,664			0		
Deionised water	0			222,216		
Electricity	4,787,251	738,363		8,701,299	850,820	
Fixed Opex	3,090,388	1,634,409		6,626,205	1,723,182	
**TOTAL**	**17,639,471**	**5,183,296**	**22,822,767**	**26,823,821**	**5,892,350**	**32,716,171**

Table S4

Unitary cost of production (UCOP) for the plasma- and electrolysis-based plants by sections

Section	Cost type	UCOP (US$/t CO)	
		Plasma-based	***Electrolysis-based***
CO_2 conversion	Capex	156	266
	Opex	363	523
Separation section	Capex	83	98
	Opex	70	76
	**TOTAL**	**671**	**962**

Table S5

Unitary cost of production (UCOP) for the plasma- and electrolysis-based plants by sections and scenarios

This table contains the underlying data for Figures 4 (base case) and 5 (scenarios) in the main paper.

	SCENARIO	Base case		(1) Equipment cost				(2) Electricity cost				(3) CO_2 feed cost				(4) All feedstock costs				(5) Conversion energy cost				(6) SPC			
	Sub-scenario			- 50%		+50%		- 50%		+50%		- 50%		+50%		- 50%		+50%		- 50%		+50%		- 50%		+50%	
	Technology	**Plasm.**	**Electr.**	**Plasm.**	**Electr.**	**Plasm.**	**Electr.**	**Plasm.**	**Electr.**	**Plasm.**	**Electr.**	**Plasm.**	**Electr.**	**Plasm.**	**Electr.**	**Plasm.**	**Electr.**	**Plasm.**	**Electr.**	**Plasm.**	**Electr.**	**Plasm.**	**Electr.**	**Plasm.**	**Electr.**	**Plasm.**	**Electr.**	**Plasm.**	**Electr.**
**Section**	**Item**													*UCOP (US$/t CO)*															
CO_2 conversion	CAPEX	156.3	266.2	78.1	133.1	234.3	399.3	156.2	266.2	156.2	266.2	156.2	266.2	156.2	266.2	156.2	266.2	156.2	266.2	78.1	532.4	234.3	177.5	208.3	266.2	208.3	266.2
	CO_2 feed	33.4	65.4	33.4	65.4	33.4	65.4	33.4	65.4	33.4	65.4	16.7	32.7	50.1	98.1	16.7	32.7	50.1	98.1	33.4	65.4	33.4	65.4	35.4	67.9	32.7	64.6
	Electricity	140.8	255.9	140.8	255.9	140.8	255.9	70.4	128.0	211.2	383.9	140.8	255.9	140.8	255.9	70.4	128.0	211.2	383.9	70.4	128.0	211.2	383.9	187.7	255.9	187.7	255.9
	Water	0.0	6.5	0.0	6.5	0.0	6.5	0.0	6.5	0.0	6.5	0.0	6.5	0.0	6.5	0.0	3.3	0.0	9.8	0.0	6.5	0.0	6.5	0.0	6.5	0.0	6.5
	Solid carbon	97.4	0.0	97.4	0.0	97.4	0.0	97.4	0.0	97.4	0.0	97.4	0.0	97.4	0.0	48.7	0.0	146.1	0.0	97.4	0.0	97.4	0.0	97.4	0.0	97.4	0.0
	Fixed Opex	90.9	194.9	52.3	106.6	125.8	279.6	90.8	194.9	90.8	194.9	90.8	194.9	90.8	194.9	90.8	194.9	90.8	194.9	52.3	363.0	125.8	136.7	110.1	190.7	116.5	196.9
Separation	CAPEX	82.7	97.6	82.7	97.6	82.7	97.6	82.7	97.6	82.7	97.6	82.7	97.6	82.7	97.6	82.7	97.6	82.7	97.6	82.7	97.6	82.7	97.6	129.4	152.8	64.0	75.6
	Electricity	21.7	25.0	21.7	25.0	21.7	25.0	10.9	12.5	32.6	37.5	21.7	25.0	21.7	25.0	10.9	12.5	32.6	37.5	21.7	25.0	21.7	25.0	40.3	48.8	15.5	17.1
	Fixed Opex	48.1	50.7	55.4	57.4	44.4	47.6	48.1	50.7	48.1	50.7	48.1	50.7	48.1	50.7	48.1	50.7	48.1	50.7	55.4	45.8	44.4	54.4	68.4	76.9	35.8	39.8
	Δ%	-	-	*-16%*	*-22%*	*16%*	*22%*	*-12%*	*-15%*	*12%*	*15%*	*-3%*	*-3%*	*2%*	*3%*	*-22%*	*-18%*	*22%*	*18%*	*-27%*	*31%*	*27%*	*-2%*	*31%*	*11%*	*13%*	*-4%*
	**TOTAL UCOP**	**671**	**962**	**562**	**748**	**780**	**1,177**	**590**	**822**	**752**	**1,103**	**654**	**930**	**688**	**995**	**524**	**786**	**818**	**1,139**	**491**	**1,264**	**851**	**947**	**877**	**1,066**	**758**	**923**

Table S6

Sensitivity analysis: Fig. 6(a) Electricity cost vs UCOP of CO

Δ%	Electricity cost ($/kWh)	UCOP (US$/t CO)	
		Plasma-based	*Electrolysis-based*
-83	0.005	536	728
-67	0.010	563	775
-50	0.015	590	822
-33	0.020	617	869
-17	0.025	644	915
0	0.030	671	962
17	0.035	698	1,009
33	0.040	725	1,056
50	0.045	752	1,103
67	0.050	779	1,150
83	0.055	807	1,196
100	0.060	834	1,243

Table S7

Sensitivity analysis: Fig. 6(b) CO_2 conversion equipment cost vs UCOP of CO

Δ%	Plasma system (US$/kW)	Electrolyser system (US$/m^2)	UCOP (US$/t CO)	
			Plasma-based	*Electrolysis-based*
-91	124	874	471	570
-80	284	2,000	496	619
-70	427	3,000	518	662
-60	569	4,000	540	705
-50	711	5,000	562	748
-40	853	6,000	584	790
-30	995	7,000	605	833
-20	1,138	8,000	627	876
-10	1,280	9,000	649	919
0	1,422	10,000	671	962
10	1,564	11,000	693	1,005
20	1,706	12,000	715	1,048
30	1,849	13,000	737	1,091
40	1,991	14,000	759	1,134
50	2,133	15,000	780	1,177
60	2,275	16,000	802	1,220
70	2,417	17,000	824	1,263
80	2,560	18,000	846	1,306
90	2,702	19,000	868	1,349
100	2,844	20,000	890	1,392

Table S8

Sensitivity analysis: Fig. 7(a) Capacity factors vs UCOP of CO

Δ%	Production (t CO/y)	Capacity factor	UCOP (US$/t CO)		Opex ratio (%)	
			Plasma-based	*Electrolysis-based*	*Plasma-based*	*Electrolysis-based*
-89	3,650	0.10	3,651	5,868	39	42
-78	7,300	0.20	1,992	3,130	44	46
-68	10,950	0.30	1,439	2,218	48	49
-57	14,600	0.40	1,153	1,752	52	52
-46	18,250	0.50	989	1,480	55	54
-35	21,900	0.60	873	1,292	58	56
-25	25,550	0.70	796	1,164	60	58
-14	29,200	0.80	733	1,062	62	60
0	34,000	0.93	671	962	64	62

Table S9

Sensitivity analysis: Fig. 7(b) CO_2 conversion equipment lifespan vs UCOP of CO

Δ%	Lifespan (years)	UCOP (US$/t CO)		Opex ratio (%)	
		Plasma-based	*Electrolysis-based*	*Plasma-based*	*Electrolysis-based*
-90	2	1,281	2,002	34	30
-80	4	934	1,411	46	42
-70	6	820	1,216	53	49
-60	8	764	1,121	57	53
-50	10	731	1,065	59	56
-40	12	710	1,029	61	58
-30	14	695	1,004	62	60
-20	16	685	986	63	61
-10	18	677	972	64	62
0	20	671	962	64	62
